# Rosiglitazone Ameliorates Spinal Cord Injury *via* Inhibiting Mitophagy and Inflammation of Neural Stem Cells

**DOI:** 10.1155/2022/5583512

**Published:** 2022-01-04

**Authors:** Qingqi Meng, Zhiteng Chen, Qingyuan Gao, Liqiong Hu, Qilong Li, Shutai Li, Lili Cui, Zhencheng Feng, Xingliang Zhang, Shiyun Cui, Haifeng Zhang

**Affiliations:** ^1^Department of Orthopedics, Guangzhou Red Cross Hospital, Jinan University, Guangzhou 510220, China; ^2^Department of Cardiology, Sun Yat-sen Memorial Hospital, Sun Yat-sen University, Guangzhou 510120, China; ^3^Department of Intensive Care Unit, Guangzhou Red Cross Hospital, Jinan University, Guangzhou 510220, China; ^4^Guangdong Key Laboratory of Age-Related Cardiac and Cerebral Diseases, Institute of Neurology, The Affiliated Hospital of Guangdong Medical University, Zhanjiang 524001, China; ^5^Institute of Pediatrics, Department of Pediatric Surgery, Shenzhen Children's Hospital, Shenzhen 518038, China; ^6^Department of Pediatrics, The Affiliated Hospital of Guangdong Medical University, Zhanjiang 524001, China; ^7^Department of Oncology, The First Affiliated Hospital of Nanjing Medical University, Nanjing 210029, China; ^8^Laboratory & Medical Research Center, Sun Yat-sen University, Guangzhou 510275, China

## Abstract

**Background:**

Neurodegenerative diseases, such as Alzheimer's disease, and traumatic brain and spinal cord injury (SCI) are prevalent in clinical practice. Inhibition of hyperactive inflammation and proliferation of endogenous neural stem cells (NSCs) is a promising treatment strategy for SCI. Our previous studies demonstrated the beneficial effects of rosiglitazone (Rosi) on SCI, but its roles in inflammation inhibition and proliferation of NSCs are unknown.

**Methods:**

SCI in a rat model was established, and the effects of Rosi on motor functions were assessed. The effects of Rosi on NSC proliferation and the underlying mechanisms were explored in details.

**Results:**

We showed that Rosi ameliorated impairment of moto functions in SCI rats, inhibited inflammation, and promoted proliferation of NSCs *in vivo*. Rosi increased ATP production through enhancing glycolysis but not oxidative phosphorylation. Rosi reduced mitophagy by downregulating PTEN-induced putative kinase 1 (PINK1) transcription to promote NSC proliferation, which was effectively reversed by an overexpression of PINK1 *in vitro*. Through KEGG analysis and experimental validations, we discovered that Rosi reduced the expression of forkhead box protein O1 (FOXO1) which was a critical transcription factor of PINK1. Three FOXO1 consensus sequences (FCSs) were found in the first intron of the PINK1 gene, which could be potentially binding to FOXO1. The proximal FCS (chr 5: 156680169–156680185) from the translation start site exerted a more significant influence on PINK1 transcription than the other two FCSs. The overexpression of FOXO1 entirely relieved the inhibition of PINK1 transcription in the presence of Rosi.

**Conclusions:**

Besides inflammation inhibition, Rosi suppressed mitophagy by reducing FOXO1 to decrease the transcription of PINK1, which played a pivotal role in accelerating the NSC proliferation.

## 1. Introduction

Neurodegenerative diseases are chronic central nervous system diseases caused by the loss of neurons or their myelin sheath. Common examples include Alzheimer's disease and traumatic brain or spinal cord injury (SCI) [1–3]. SCI is a devastating medical condition that leads to the deterioration of health and substantial consumption of healthcare resources [4]. The initial neural tissue disruption during SCI can trigger secondary damage and extensive inflammatory reactions, leading to cell death at the injury site. Also, SCI triggers the active form of proinflammatory cytokines such as interleukin-1 beta (IL-1*β*) to initiate neuroinflammation [5, 6]. SCI can be a complication of numerous insults (e.g., trauma and compression for various reasons), leading to new neurologic damage. It is associated with a poor prognosis, and thousands of individuals are affected by this condition each year. Therefore, SCI is one of the most severe neurologic disorders, and its treatment has been a significant challenge.

Following SCI, transplanting neural stem cells (NSCs)/neural progenitor cells (NPCs) is a promising strategy to enhance neuro-regeneration. Unfortunately, the survival of grafted NSCs/NPCs after SCI is dissatisfactory. Therefore, it is crucial to develop new methods to increase the survival of NSCs/NPCs [7]. The repair of SCI largely depends on the proliferation of NSCs/NPCs [8, 9]. In mice, the lack of spinal cord NSCs exacerbates proinflammatory cytokine expression and cripples neuronal cell survival and functional recovery [10]. Although many studies have explored effective strategies to accelerate the proliferation of NSCs, only a few of them have been of practical importance [11]. Among the strategies with potential clinical significance, thiazolidinediones (TZDs) may be the most promising. TZDs, a class of heterocyclic compounds consisting of a five-membered C_3_NS ring, are classical antidiabetic drugs. They ameliorate insulin resistance by activating peroxisome proliferator-activated receptor-gamma (PPAR-*γ*). Rosiglitazone (Rosi) is one of the extensively used TZDs, and our previous study demonstrated its role in improving limb function in SCI rats [12]. However, the underlying mechanisms are still elusive.

Considering the great importance of NSC proliferation in SCI therapies, there were some preliminary explorations about the role of TZDs on it. Some studies have reported the effects of TZDs on mitochondrial functions, which are vital for stem cell survival and proliferation. For example, Rosi protects the NSCs against various toxicities *via* mitochondria-related inflammation and apoptosis [13–15]. Mitophagy is an essential biological process that modulates mitochondria-related oxidation and inflammation [16–18]. The maturation of mitophagy increases fatty acid utilization and decreases glucose utilization [19], which often implies a reduction in proliferation. This is particularly important for NSCs because they are highly dependent on glycolysis for maintaining their growth and homeostasis [20, 21]. Indeed, mitophagy is associated with the repressed proliferation of some stem cells and malignant cells [22, 23]. The association between TZDs and mitophagy is unknown. Therefore, we hypothesized that Rosi regulates mitophagy and switches substrate metabolism, thereby promoting the proliferation of NSCs and accelerating the repair of SCI. We first demonstrated the effects of TZDs on NSC proliferation under SCI and the role of mitophagy in that process. Subsequently, we explored the detailed mechanism by which Rosi regulates mitophagy.

## 2. Materials and Methods

### 2.1. Animal Treatments and Cell Cultures

All of the animal experiments complied with the ARRIVE guidelines. They were conducted in accordance with the National Institutes of Health Guide for the Care and Use of Laboratory Animals (NIH Publication No. 8023, revised 1978). SCI was induced in animals as per previous reports. Briefly, 12-week-old male Sprague–Dawley (SD) rats were housed at room temperature in a 12-hour light/dark cycle, and they were allowed access to chow diet and water *ad libitum*. The rats were anesthetized and dropped using an impactor (weight: 10 g; diameter: 2.5 mm) from a height of 25 mm to induce SCI. Afterward, T_9_ to T_10_ laminectomy was performed. SCI rats were randomly assigned to the Rosi- and the DMSO-treated groups, which then intragastrically received Rosi (3 mg/kg/day, GSK, Middlesex, United Kingdom) or the same volume of saline, respectively. Rosi was dissolved in DMSO as stock solution, which was diluted with saline for experiments. After 8 weeks of treatment, motor function recovery from SCI was assessed using the Basso–Beattie–Bresnahan locomotor rating scale (BBB scale). The rats were killed, and then, the injured spinal cords (centered on the lesion point) from SCI model rats were harvested for subsequent assays. Especially, before the spinal cord was harvested for flow cytometry, 5-bromo-2′-deoxyuridine (BrdU, 50 mg/kg/day) was intraperitoneally delivered into rats one time for seven consecutive days.

NSCs were cultured as described in our previous report [12]. Briefly, NSCs were obtained from the spinal cord of newborn SD rats (within 24 hours). Dissociated cells were suspended in DMEM/F12 medium (high glucose, Thermo Fisher Scientific, Massachusetts, USA) supplemented with 10% fetal bovine serum (Thermo Fisher Scientific, Massachusetts, USA), 2% B27 (Thermo Fisher Scientific, Massachusetts, USA), 20 *μ*g/L epidermal growth factor (Sigma, Missouri, USA), 20 *μ*g/L base fibroblast growth factor (Sigma, Missouri, USA), 50 *μ*mol/L 2-mercaptoethanol (Sigma, Missouri, USA), 0.5 mmol/L L-glutamine (Thermo Fisher Scientific, Massachusetts, USA), and 1% streptomycin and penicillin (Thermo Fisher Scientific, Massachusetts, USA). NSCs were seeded 12 hours before treatments. Rosi (Cayman Chemical, Michigan, USA) was used at the indicated concentration.

To avoid the influence of other cells in spinal cord, NSCs in saline or Rosi-treated rats were isolated by antinestin magnetic beads. The dissociation solution consisted of 150 U/mL collagenase type 1, 1 mg/mL trypsin, and 15 U/mL DNaseI in the Krebs-Henseleit buffer. The isolated cells were resuspended in 1 : 100 antinestin beads and incubated for 15 min at 4°C. After washing nestin antibody-bound cells by adding equilibration buffer, the cells were resuspended in 100 *μ*L of DMEM/F12 medium containing magnetic bead–conjugated goat anti-rabbit beads and were incubated for 15 min at 4°C. Subsequently, 2 mL of culture medium was added to the tube that was placed in the magnetic separator. The supernatant containing cells that had not bound magnetic beads was collected, and 2 mL culture medium was added to the tube. The magnet was then removed, and the cells that had bound magnetic beads were collected. Magnetically separated nestin postive fibroblasts can be used for PCR, western blot, and other downstream applications.

For the detection of PTEN induced putative kinase 1 (PINK1) and forkhead box protein O1 (FOXO1), NSCs were treated with Rosi for 48 hours. For the dual-luciferase reporter gene assay and chromatin immunoprecipitation (CHIP), Rosi was administered after 18 hours of induction of FOXO1 using doxycycline (DOX) dissolved in phosphate-buffered saline (PBS). Afterward, NSCs were cultured and incubated in tetracycline-free serum in the presence of Rosi or the DMSO control for 24 hours. Carbonyl cyanide 3-chlorophenylhydrazone (CCCP, Sigma, Missouri, USA) and actinomycin D (ActD, 1 *μ*g/mL, Sigma, Missouri, USA) were used at the indicated concentrations and time points.

### 2.2. Plasmid Construction and Lentivirus Packaging

Lentivirus plasmids expressing mitoTimer, FOXO1, and PINK1 were constructed. The mitoTimer and PINK1 were gifts from Roberta Gottlieb (Addgene plasmid, 50547) and Mark Cookson (Addgene plasmid, 13323), respectively. FOXO1 was synthesized *in vitro*. To avoid the disadvantages caused by a consecutive expression, the coding sequence for each of the three genes was cloned into the tetracycline-induced expression backbone vector (pLVX-TetOne-Puro, Clontech, Otsu, Japan) using incision enzymes *Eco*R I/*Bam*H I (mitoTimer), *Age* I/*Eco*R I (PINK1), and *Bam*H I/*Bst*Z1107 I (FOXO1). The constructed vectors were confirmed by DNA sequencing.

For lentivirus packaging, packaging vectors (psPAX2 and pMD2.G) and expression vectors (pLVX-TetOne-Puro-mitoTimer/FOXO1/PINK1) were cotransfected into 293T cells using FuGene transfection reagents. Titers of the pseudovirus were determined by PCR, and a known titer of the lentivirus was used as the control. Primers targeting the long terminal repeat were used in PCR. A multiplicity of infection (MOI) of 30 was used in all of the experiments. The indicated concentration of DOX was used to induce expression.

### 2.3. CCK-8 Measurement, ATP Production, Edu Staining, Cell Counting, and Glucose and Lactic Acid Assays

The proliferation of NSCs was assessed by the Cell Counting Kit-8 (CCK-8, Dojindo Molecular Technologies, Kumamoto, Japan), adenosine triphosphate (ATP) production (Biovision Inc., California, USA), 5-ethynyl-2′-deoxyuridine (Edu) (Ribobio, Guangzhou, China) incorporation, and cell counting. After treated with DMSO or Rosi for 18 hours, 24 hours, and 36 hours, proliferation of NSCs was assessed by CCK-8, ATP production, and Edu incorporation, respectively. CCK-8 and ATP production were measured according to the instructions of the manufacturers. Edu is a thymidine analogue, which can replace thymine to infiltrate the replicating DNA molecules during cell proliferation. The Edu-binding fluorescent dye accurately reflects cell proliferation. The cells were collected and washed with PBS three times for subsequent Edu and nuclear staining according to the manufacturer's instructions. Cells were collected and resuspended for counting using Countstar (ALIT Life Science Co. Limited, Shanghai, China).

For intracellular glucose assessment, cells were treated with DMSO or Rosi for 12 hours in DMEM medium. Afterward, cells were ultrasonically fragmented in glucose-free PBS and centrifuged. Glucose levels in the supernatants were determined using a commercially available kit (Biovision Inc., California, USA). Total protein levels in the precipitation were determined and used for normalization. For the lactic acid assay, cells were subjected to the same treatments for 24 hours, and both the supernatants and cells were collected. Lactic contents in the supernatant were determined using a biochemical analyzer. Cells were counted, and lactic acid concentration per cell was calculated.

### 2.4. Biochemical Detections

Cells were treated in the complete medium as indicated, and they were subjected to total or mitochondrial protein extraction using the respective kits. Protein concentration was determined by the Bradford protein assay. Extracted proteins were then resolved on SDS-PAGE, and they were transferred to a polyvinylidene fluoride membrane. Blots were incubated with the antibodies for FOXO1 (Cell Signaling Technology, Massachusetts, USA), PINK1 (Abcam, Massachusetts, USA), *β*-actin (Cell Signaling Technology, Massachusetts, USA), Complex I (NDUFB8, Cell Signaling Technology, Massachusetts, USA), Complex IV (mt-CO1, Cell Signaling Technology, Massachusetts, USA), FUNDC1 (Abcam, Massachusetts, USA), Bnip3 (Abcam, Massachusetts, USA), and Nix (Cell Signaling Technology, Massachusetts, USA) or voltage-dependent anion-selective channel 1 (VDAC1, Cell Signaling Technology, Massachusetts, USA) at 4°C overnight. Afterward, bound antibodies were visualized using peroxidase-coupled secondary antibodies and ECL reagents (Thermo Fisher Scientific, Massachusetts, USA). The bands of interest were quantified using the Image J densitometry scanning program. For Western blots of complexes I and IV and VDAC1, total mitochondrial proteins were isolated using the commercially available kit (Qiagen, Duesseldorf, German). *β*-Actin and VDAC1 were used as the loading control for total proteins and mitochondrial proteins, respectively.

The productions of IL-1*β* (BMS630, Invitrogen) and IL-6 (BMS625, Invitrogen) in the primary NSCs from SCI rats were measured by enzyme-linked immunosorbent assay (ELISA) according to the manufacturer's instructions.

### 2.5. Real-Time RT-PCR Assays

NSCs were grown in 6-well plates. Total RNA was isolated using TRIZOL reagent (Takara, Tokyo, Japan). The integrity and purity of total RNA were electrophoretically and spectroscopically confirmed. Reverse transcription of 800 ng of total RNA was performed with a commercially available kit (Takara, Tokyo, Japan) using hexamer primers. Detailed procedures were performed according to the manufacturer's protocol. Real-time PCR was performed on a LightCycler® 96 System (Roche, Basel, Switzerland) with SYBR® Premix Ex Taq™ II (Takara, Tokyo, Japan). Instead of the first-strand cDNA, water was used as the negative control, and primers using plasmid-containing genes of interest were used as positive controls. *β*-Actin mRNA levels were used to normalize each sample and gene. All of the primers used in real-time RT-PCR are listed in Table S1–3.

### 2.6. Mitophagy and Oxygen Consumption Detection

Mitophagy was assessed by colocalization of LC3B and the mitochondrial marker (Tomm20) stained by immunofluorescence. Cells were grown on a glass slide in 6-well plates with the indicated treatments. For immunofluorescence, cells were washed and fixed in 4% paraformaldehyde. Cell membranes were permeabilized with 1% Triton. LC3B (Abcam, Massachusetts, USA) and Tomm20 (Abcam, Massachusetts, USA) antibodies were incubated for 12 hours. Alexa 488-labeled rabbit IgG antibody and Dylight 549-labeled mouse IgG antibody were incubated. Finally, cell nuclei were restained with DAPI and examined by confocal imaging (LSM70, Carl Zeiss, Oberkochen, Germany).

The mitoTimer is modified from a fluorescent timer, in which fluorescence shifts over time from green to red as the protein matures. It is a tool for monitoring mitochondrial turnover [24]. Enhanced mitophagy accelerates mitochondrial clearance, which results in the reduction of red fluorescence. Besides, mitoTimer can only be imported to the healthy mitochondria. Therefore, enhancement of green fluorescence indicated an increase in healthy mitochondrial biogenesis. Doxycycline (3 *μ*g/mL, MP Biomedicals, Ohio, USA) was used to induced mitoTimer expression 24 hours before treatments, and the results were observed under confocal fluorescence microscopy.

For the oxygen consumption test, cells were treated with DMSO or Rosi for 48 hours, and the medium was removed. According to the manufacturer's instructions, oxygen consumption was detected using a commercially available kit (Abcam, Massachusetts, USA). Succine and adenosine diphosphate (ADP) were used to induce the state 2 and state 3 respiration, respectively.

### 2.7. Flow Cytometry

The proportion of Nestin- and BrdU-positive (Nestin^+^/BrdU^+^) cells in the spinal cord of SCI rats was measured by flow cytometry. Rats were anesthetized, and the spinal cords were removed, which were then subjected to trypsin and collagenase for digestion of the matrix, and single-cell suspensions were prepared. Cells were incubated with the primary antibodies against Nestin (Abcam, Massachusetts, USA) and BrdU (Abcam, Massachusetts, USA), followed by a FITC- or Cy3-labeled secondary antibody. Subsequently, cells were washed and resuspended in PBS, and they were subjected to flow cytometry analysis. Auto-fluorescence and isotype controls were used routinely as controls.

Mitochondria were stained with mitoTracker Green (Thermo Fisher Scientific, Massachusetts, USA), together with mitoTracker Red (Thermo Fisher Scientific, Massachusetts, USA) or mitoSOX (Thermo Fisher Scientific, Massachusetts, USA). mitoTracker Green stains all of the mitochondria independent of the membrane potential, while mitoTracker Red accumulation in the mitochondria relies on the normal membrane potential. mitoSOX serves as a mitochondrial reactive oxygen species (ROS) probe. NSCs were incubated with mitoTracker Green/Red or mitoTracker Green/mitoSOX (concentrations of all agents were 100 nmol/L) for 15 min, followed by washing twice with PBS to remove the unbound dyes. Cells were collected and resuspended in PBS. Finally, cells were subjected to flow cytometry analysis. All flow cytometry data were analyzed using the FlowJo software X (Oregon, USA). The unstained cells were used to set the gates in the FlowJo program.

### 2.8. Bioinformatic Analysis, Dual-Luciferase Reporter Gene Assay, Site-Directed Mutagenesis, and Chromatin Immunoprecipitation (CHIP)

Bioinformatic analysis, dual-luciferase reporter gene assay, site-directed mutagenesis, and chromatin immunoprecipitation were used to identify the FOXO1 binding sites in the PINK1 gene. Except for the gene promoter miner (GPMiner), which was performed according to another study [25], all other assays and analyses were performed as per our previous report [26].

Promoter region sequences were determined to search the potential transcription factor-binding sites. Putative core regulatory elements (e.g., GC boxes, INR, CAAT boxes, or TATA boxes) were bioinformatically analyzed by the Genomatix MatInspector [26] (Transcription factor binding sites, weight matrices; Matrix Library Version 9.4; Core similarity of 0.75 and Matrix similarity of “Optimized”) and GPMiner [25], respectively. The predictive sequence that regulates PINK1 transcription was validated by the dual-luciferase reporter gene assay. Segments with the indicated region in the *PINK1* gene were cloned into pGL3-Basic vectors. To better demonstrate that Rosi repressed PINK1 transcription through FOXO1, we used DOX (1 *μ*g/mL) to induce FOXO1 expression in NSCs. NSCs were electroporated (1 × 10^4^ cells; voltage: 250 V; capacitor: 600 *μ*F; plasmid: 1 *μ*g, respectively, for each) to import pGL3 and pRL-TK and seeded in 96-well plates. Cells were cultured for 24 hours after electroporation. Detection procedures were performed according to the manufacturer's protocol. Luciferase activities were normalized to the cotransfected pRL-TK plasmid.

Site-directed mutagenesis was performed using the commercially available QuikChange®-II kit (Agilent Technologies, California, USA). Briefly, PCR was performed to obtain the amplicon containing the desired nucleotide mutations using the nonmutated plasmid as the template. PCR products were purified and subjected to *Dpn* I digestion to erase the unmutated plasmid, and the cells were then transformed into competent cells. The mutated plasmid was sequenced confirmed. All of the primers used are listed in Supplementary materials.

CHIP was performed using the Pierce Agarose CHIP Kit (Thermo Fisher Scientific, Massachusetts, USA) as per our previous report [26]. Cells were plated in a 10 cm dish with the indicated treatments. The medium was removed, and the cells were washed with precooled PBS. Cells were cross-linked in 1% formaldehyde and resuspended, followed by sequential lysis of the cells and nuclear membrane. After lysis, sonication (8 seconds with a 10-second intermission for six times) was performed to generate sheared DNA fragments. Supernatants were collected and subjected to agarose protein A/G to remove the nonspecific bound DNA. Agarose was removed, and supernatants, whose 10% volume was collected for the input, were collected for chromatin immunoprecipitation with the FOXO1 antibody overnight at 4°C followed by the new agarose protein A/G. The supernatants were removed, and agarose was collected for elution and reverse-cross linking to recover the bound DNA. Finally, the recovered DNA was purified and used as the template for PCR detection using primers flanking the FOXO1 binding sites in the first intron of the *PINK1* gene. Primers' sequences are listed in Supplementary Tables.

### 2.9. Statistical Analysis

Data are expressed as the mean ± SEM. Statistical significance among groups was determined by using the *t*-test (the unpaired *t*-test was used for the independent groups, while the paired *t*-test was used for assessing the preoperation and postoperation JOA scores in the clinical data) or one-way ANOVA followed by the SNK test for multiple comparisons if three or more groups were involved. The Chi-square test was used to test for equality of proportions between populations. Logistic regression was used to measure the influence of the independent variables (age, gender, HbA1c, and Rosi/no-Rosi treatment) on the dependent variable (mean difference in the JOA score between the preoperation and postoperation categories was as follows: H-JOAIR, mean difference in JOA > 2; otherwise, L-JOAIR). Visualized results (nomogram) were obtained. A nomogram was produced by R-statistics version 3.5.0 with the RMS package [27], and the remaining statistical graphs were created by the GraphPad Prism Software (California, USA).

## 3. Results

### 3.1. Rosi Ameliorates Spinal Cord Injury, Inhibits Inflammation Factors, and Promotes Glucose Utilization for ATP Production in the Proliferation of NSCs

**Figure 1:**
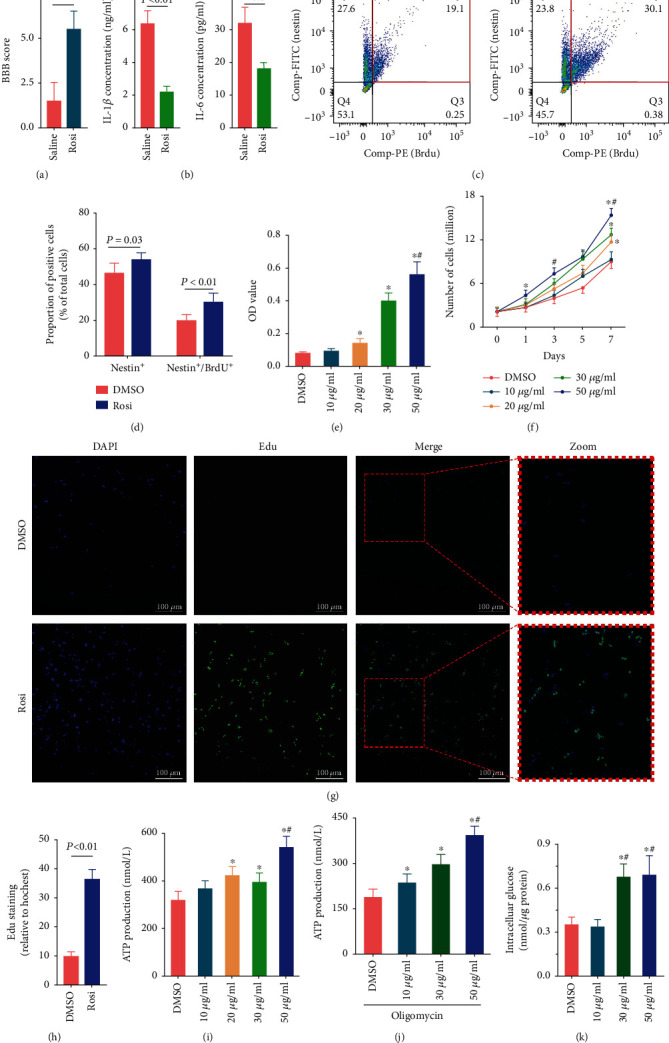
Rosi ameliorates spinal cord injury, inhibits inflammation factors, and promotes NSC proliferation. (a) BBB scores in SCI rats with or without Rosi treatment. (b) The levels of IL-1*β* and IL-6 in NSCs by ELISA kit. (c) Representative plots of flow cytometry. The Nestin^+^/BrdU^+^ cells in the red rectangle are those of interest. (d) Flow cytometry results showing Nestin^+^ cells and Nestin^+^/BrdU^+^ cells in the spinal cord of SCI rats undergoing DMSO or Rosi treatments. (e, f) CCK-8 measurement and cells counting in NSCs treated with DMSO or different concentrations of Rosi. ^#^*P* < 0.05 versus 30 *μ*g/mL Rosi. (g, h) Images of Edu staining in NSCs treated by DMSO or Rosi. DAPI (blue) and Edu (green) represent the nucleus and the newly synthesized DNA, respectively. (i) Effects of Rosi on ATP production. ^#^*P* < 0.05 versus 30 *μ*g/mL Rosi. (j) Effects of Rosi on ATP levels produced by glycolysis. ^#^*P* < 0.05 versus 30 *μ*g/mL Rosi. (k) Effects of different Rosi concentrations on intracellular glucose content. ^#^*P* < 0.05 versus 20 *μ*g/mL Rosi. Data represent six (a) or three (b, c, e–g, i–k) independent experiments and are expressed as mean ± SEM (a–c, e–g, i–k). ^∗^*P* < 0.05 versus DMSO treatment.

To assess the effects of Rosi treatment on the recovery of spinal cord function after SCI, we compared the BBB scores between the saline- and Rosi-treated rats. The score was dramatically higher in rats treated with Rosi (Figure 1(a)), indicating that Rosi accelerated SCI recovery. To investigate the change of inflammation of primary NSCs from SCI rats in response to Rosi treatment, the expression levels of inflammatory cytokines were examined by ELISA. The expression levels of IL-1*β* and IL-6 in the Rosi group were significantly lower than those in the control group (Figure 1(b)).

Given that proliferation of NSCs is crucial for the recovery of SCI, we examined the proliferation of NSCs in the spinal cord of SCI rats treated with saline or Rosi by flow cytometry. Primary NSCs were isolated from rats receiving saline or Rosi treatment, and proportions of Nestin positive (Nestin^+^) and/or BrdU positive (BrdU^+^) cells were determined. Nestin is a characteristic marker of NSCs. BrdU is a thymine derivative, which can stably integrate into the replicating DNA instead of thymine (T). Thus, a BrdU-specific antibody can detect cell proliferation. The proportion of Nestin positive cells (including Nestin^+^/BrdU^−^ and Nestin^+^/BrdU^+^, 53.68 ± 4.19%) in Rosi-treated rats was increased by 15.86% in comparison with the saline-treated group (46.33 ± 5.67%). Nestin and BrdU double-positive (Nestin^+^/BrdU^+^) cells in Rosi-treated group were increased by 0.54-fold compared to the control group (30.04 ± 5.17% vs. 19.46 ± 4.03%; *P* < 0.01) (Figures 1(c) and 1(d)). These data indicated that Rosi not only increased the number of NSCs but that it also promoted the proliferation of NSCs. To further confirm the increased proliferation of NSCs upon Rosi administration, we investigated the effects of Rosi on NSCs *in vitro* by CCK-8, cell counting, and Edu staining. The Rosi administration tended to increase the NSCs viability in a dose-dependent manner, as shown by CCK-8. The 20 *μ*g/mL group, particularly the 30 *μ*g/mL and 50 *μ*g/mL groups, showed a significant increase in NSC viability compared with the control group (Figure 1(e)). The results from cell counting showed that Rosi treatment resulted in a 1.5-fold increase in NSCs on day 7; moreover, it shortened the doubling time to approximately 48 hours, which was even shorter (within 24 hours) at higher concentrations of Rosi treatment (Figure 1(f)). Given that DNA replication is necessary for cell proliferation, we finally assessed cell proliferation by Edu staining assay. The proportion of Edu positive-NSCs significantly increased after Rosi treatment (Figures 1(g) and 1(h)).

Considering that ATP is essential for cell proliferation, we explored changes of its production in response to Rosi. As shown in Figure 1(i), compared with the control group, ATP production upon Rosi administration increased slightly in 20 *μ*g/mL or 30 *μ*g/mL groups and notably (1.69-fold) at 50 *μ*g/mL group. Glucose and fatty acids are the leading resources for ATP production, mainly via oxidative phosphorylation (OXPHOS) in mitochondria. The oxygen consumption assays showed that Rosi did not significantly affect OXPHOS (Figure S1A–E). Therefore, we further determined whether the ATP production enhanced by Rosi came from glycolysis. In the presence of oligomycin, an ATP synthase inhibitor during OXPHOS process, ATP production in Rosi-treated cells was much higher than that in the control group (Figure 1(j)). Notably, the amount of ATP production (approximately 230 nmol/L) in response to Rosi at 50 *μ*g/mL was higher than that in the control group regardless of oligomycin intervention (Figures 1(i) and 1(j)), indicating that the Rosi-induced increase in ATP production was mainly the result of glycolysis. Rosi at 30 *μ*g/mL and 50 *μ*g/mL significantly increased glucose uptake by NSCs (Figure 1(k)) but did not obviously alter the supernatant lactate content (Figure S2).

### 3.2. Rosi Promotes Proliferation of NSCs via Inhibition of PINK1-Mediated Mitophagy

**Figure 2:**
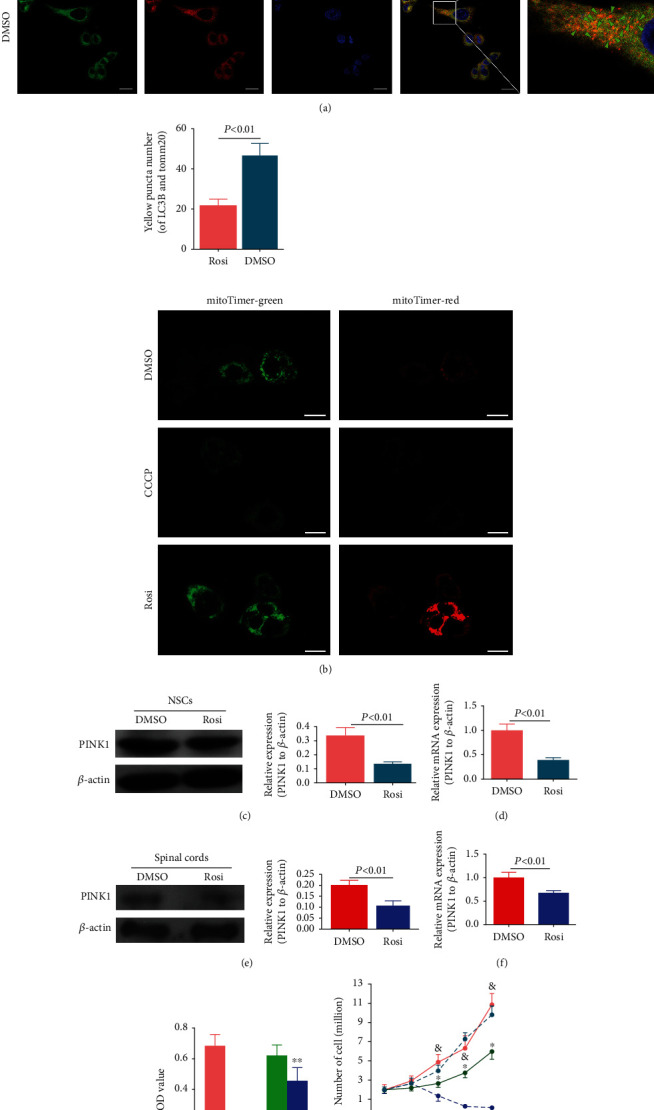
The roles of mitophagy played in Rosi-induced NSC proliferation. (a) Confocal immunofluorescence images showing LC3B (red) and Tomm20 (green) costaining in NSCs. The statistical analyses were performed base on four randomly selected scopes under the confocal microscope. Scale bar represents 10 *μ*m. (b) Confocal fluorescence images showing the fluorescence of mitoTimer. Green fluorescence represents younger and healthier mitochondria (usually less than 48 hours), while red fluorescence represents mitochondria existing for a longer time (usually longer than 48 hours). Scale bar represents 10 *μ*m. (c) Western blot images showing the effects of Rosi on PINK1 expression in Rosi-treated NSCs. (d) PCR results showing the effects of Rosi on PINK1 expression in Rosi-treated NSCs. (e) Western blot images showing the effects of Rosi on PINK1 expression in the NSCs from the injured spinal cords. (f) PCR results showing the effects of Rosi on PINK1 expression in the NSCs from the injured spinal cords. (g, h) CCK-8 measurement (g) and cell counting (h) showing effects of restored mitophagy by either CCCP administration or the forced PINK1 expression on Rosi-induced cell proliferation. Rosi was applied in all the groups in addition to the indicated treatments. Data represent three independent experiments and are expressed as mean ± SEM. ^∗^*P* < 0.05 versus DMSO treatment; ^∗∗^*P* < 0.05 versus PBS treatment.

Mitophagy has been shown to play an essential role in both cell proliferation and substrate metabolism. Therefore, we explored whether mitophagy is implicated in the proliferation of NSCs induced by Rosi. After treatment with Rosi or DMSO, NSCs were observed with laser confocal microscopy for immunofluorescence colocalization of autophagy marker LC3B and mitochondrial marker Tomm20. Rosi decreased the punctuated distribution of LC3B in the cytoplasm, which colocalized with Tomm20, indicating inhibition of mitophagy (Figure 2(a)). The mitochondria quality and state can be monitored by an indicator protein mitoTimer protein located in the mitochondrial membrane [24]. The low red/green ratio of mitoTimer indicates that mitochondria are younger and healthier. In contrast, the increase in red/green ratio indicates the aging of mitochondria, suggesting that mitophagy responsible for clearing aged mitochondria is blocked. Compared with the DMSO-treated control group, the mitophagy inducer CCCP markedly reduced the red/green ratio, which was in accordance with the clearance of mitochondria by CCCP. Nevertheless, the Rosi-treated cells showed an obvious increase in red/green ratio, indicating the delayed clearance of mitochondria (Figure 2(b)). Interestingly, green fluorescence was also increased under Rosi treatment, indicating enhanced mitochondrial biogenesis (Figure 2(b)).

There are three canonical pathways inducing mitophagy, namely, PINK1/Parkin, Bnip3L/Nix (including Bnip3), and Fundc1. However, levels of Bnip3, Nix, and Fundc1 were not significantly different between DMSO- and Rosi-treated NSCs (Figure S3). In contrast, Western blot results showed that the expressions of mitochondrial PINK1 were decreased in Rosi-treated NSCs (Figure 2(c)). Results from PCR also yielded consistent results (Figure 2(d)). Similarly, PINK1 levels in the NSCs from the injured spinal cords were decreased in rats receiving Rosi (Figures 2(e) and 2(f)).

To study the effects of the depressed PINK1 and subsequent mitophagy impairment in Rosi-induced NSC proliferation, we performed rescued experiments. To avoid the excessive burden of mitochondria caused by consecutive expression of PINK1, we adopted a tetracycline-induced expression system to control the forced expression of PINK1 in NSCs. After the inhibition of mitophagy under Rosi administration was relieved by either the forced expression of PINK1 or the mitophagy inducer CCCP, proliferation of NSCs was assessed. The levels of PINK1 inhibited by Rosi (50 *μ*g/mL) were restored only in the presence of DOX. CCK-8 and cell counting assays showed that forced PINK1 expression and CCCP administration decelerated the proliferation of NSCs (Figures 2(g) and 2(h)).

**Figure 3:**
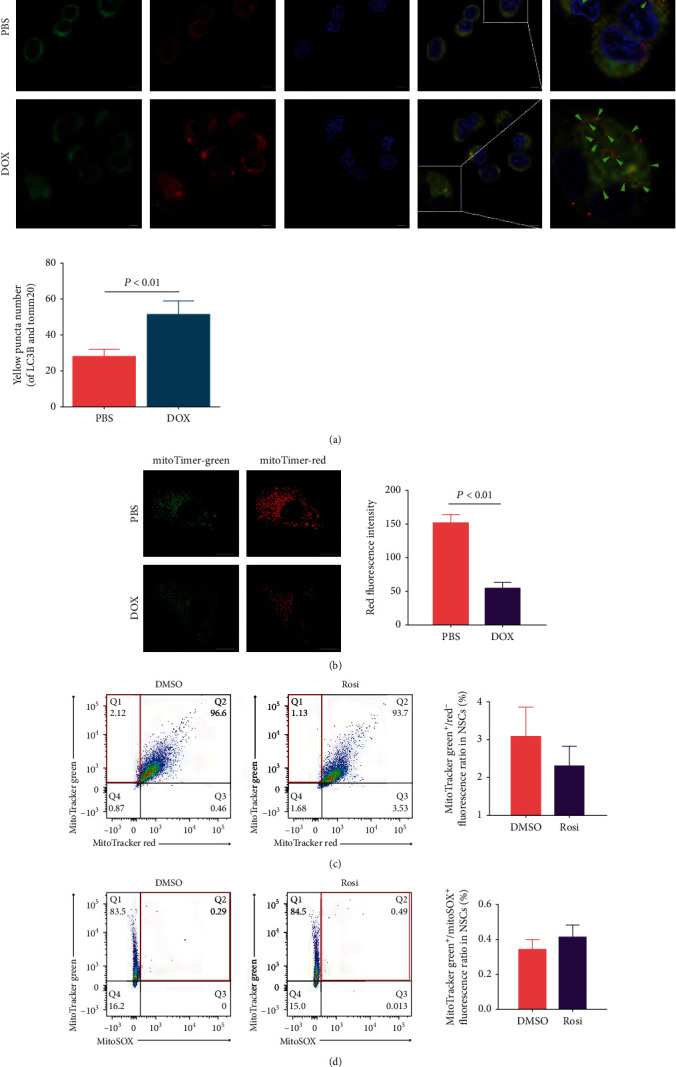
Overexpression of PINK1 counteracted relieved Rosi-induced suppression of mitophagy. (a, b) Confocal immunofluorescence images showing costaining of LC3B (red) and Tomm20 (green) (a) and mitoTimer (b) in NSCs, in which the forced PINK1 expression was induced by DOX or by the PBS as control and then subjected to Rosi. The forced PINK1 expression was controlled at the level similar to that without Rosi treatment. The statistical analyses were performed base on four randomly selected scopes under the confocal microscope. Scale bar represents 10 *μ*m. (c, d) Flow cytometry results showing mitoTracker Green^+^/mitoTracker Red^+^ cells (c) and mitoTracker Green^+^/mitoSOX^+^ cells (d) with DMSO or Rosi treatments. The cells in the red rectangle are those of concern. Data represent three independent experiments.

We next investigated the association between decreased PINK1 and inhibited mitophagy under Rosi treatment. The forced expression of PINK1 in NSCs by DOX enhanced the puncta of LC3B and the colocalization between LC3B and mitochondrial marker Tomm20 (Figure 3(a)), demonstrating the restoration of mitophagy inhibited by Rosi. In mitoTimer-expressing NSCs under Rosi treatment, compared with the vehicle control cells, the forced expression of PINK1 by DOX resulted in significantly reduced red fluorescence and slightly reduced green fluorescence, thereby verifying that the forced expression of PINK1 conspicuously reversed the suppressed mitophagy in the presence of Rosi (Figure 3(b)). Because PINK1 expression was induced up to the levels similar to those in steady-state NSCs without treatment, these results (Figures 3(a) and 3(b)) imply that excessive mitophagy may exist in NSCs. To further clarify this implication, NSCs were treated with Rosi or DMSO, and mitochondria were stained with mitoTracker Green plus mitoTracker Red or mitoSOX, which indicated healthy mitochondria and mitochondrial reactive oxygen species (ROS) contents, respectively. Flow cytometry showed that both the impaired mitochondria (mitoTracker Green^+^/mitoTracker Red^−^) and mitochondrial ROS (mitoTracker Green^+^/mitoSOX^+^) were similar between Rosi- and DMSO-treated cells (Figures 3(c) and 3(d)).

Taken together, all of the above data showed that Rosi promoted proliferation of NSCs, at least in part, via mitophagy inhibition, which was mediated by a reduction in mitochondrial PINK1. Besides, excessive mitophagy could exist in NSCs.

### 3.3. Rosi Inhibits PINK1 Expression through FOXO1 Downregulation

**Figure 4:**
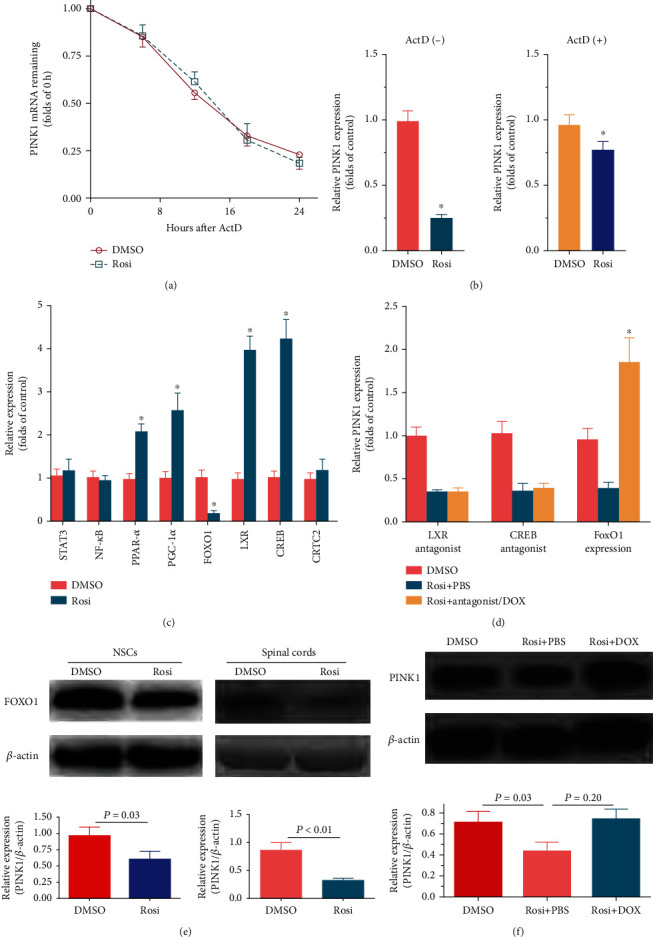
Identification of FOXO1 as a key transcription factor in Rosi-induced PINK1 suppression. (a) PINK1 mRNA stability test. DMSO or Rosi was administered to NSCs for 12 hours before ActD treatment. PINK1 mRNA was measured immediately, 8 hours, 16 hours, and 24 hours after ActD treatment. (b) Effects of ActD on PINK1 mRNA contents in cells treated by DMSO control or Rosi. The data of ActD(-) group (for both DMSO and Rosi) were normalized to PINK1 expression levels in the cells without any treatment. The data of ActD(+) group (for both DMSO and Rosi) were normalized to PINK1 expression levels in cells with ActD treatment. (c) Effects of Rosi on mRNA expression of the eight transcription factors identified to be involved in TZDs-related pathways by KEGG analysis. (d) Effects of antagonists/agonists against the mostly altered transcription factors by Rosi (LXR, CREB, and FOXO1) on PINK1 mRNA expression. (e) Western blot images showing the effects of Rosi on FOXO1 protein expression. (f) Effects of the forced FOXO1 expression on Rosi-induced PINK1 expression. *β*-Actin was used as an internal reference (a–f). The mRNA content was measured by RT-qPCR. Data represent three independent experiments and are expressed as mean ± SEM (a–d). ^∗^*P* < 0.05 versus DMSO or PBS.

The above data demonstrated that PINK1 and mitophagy played essential roles in the Rosi-induced proliferation of NSCs, and we next elucidated the underlying mechanism regulating PINK1 expression. We first used ActD, a transcription inhibitor, to prevent RNA polymerase elongation for assessing the mRNA stability to determine whether the transcriptional or posttranscriptional mechanisms were involved. The degradation rates of PINK1 RNA showed a similar pattern between the Rosi-treated group and the DMSO control group (Figure 4(a)). As shown in Figure 4(b), the mRNA level of PINK1 in NSCs upon Rosi administration was decreased by three-quarters compared with that in the control group. After NSCs were pretreated with ActD and treated with Rosi or DMSO, the mRNA level of PINK1 in NSCs after Rosi administration decreased by about one-quarter in comparison with that after DMSO administration. These results indicated that the inhibition of transcription was the primary mechanism of Rosi-induced PINK1 suppression.

To account for the Rosi-induced PINK1 transcriptional reduction involved in mitophagy inhibition, we next identified the related transcription factors of PINK1. Rosi is a specific agonist of the nuclear receptor transcription factor PPAR-*γ*. A wide range of target genes of PPAR-*γ* were subjected to enrichment analysis of the KEGG signaling pathway, but none of them were directly associated with mitophagy. Rosi as a TZD antihyperglycemic agent mainly works by improving insulin action. Rosi can increase the uptake of glucose by NSCs (Figure 1(k)). Therefore, we focused on the genes involved in the insulin and glycolysis pathways as well as mitophagy, and we found eight candidate transcription factors. Except for STAT3, NF-*κ*B, and CRTC2, the mRNA expression levels of other candidates were significantly altered by Rosi administration, among which FOXO1 mRNA was exclusively decreased (Figure 4(c)). A CREB or LXR antagonist did not abolish the effects of Rosi on PINK1 expression (Figure 4(d)). Due to the unavailability of the FOXO1 agonist, we used the tetracycline-induced system to restore FOXO1 expression. The forced FOXO1 expression reversed the decrease in PINK1 expression under Rosi (Figure 4(d)). Downregulation of FOXO1 proteins in NSCs was confirmed by western blotting (Figure 4(e)). Besides, the changes of FOXO1 levels could also be observed in the NSCs purified from Rosi-treated SCI rats (Figure 4(e)). The Rosi-induced reduction in PINK1 protein level was reversed by the forced FOXO1 expression (Figures 4(f)).

**Figure 5:**
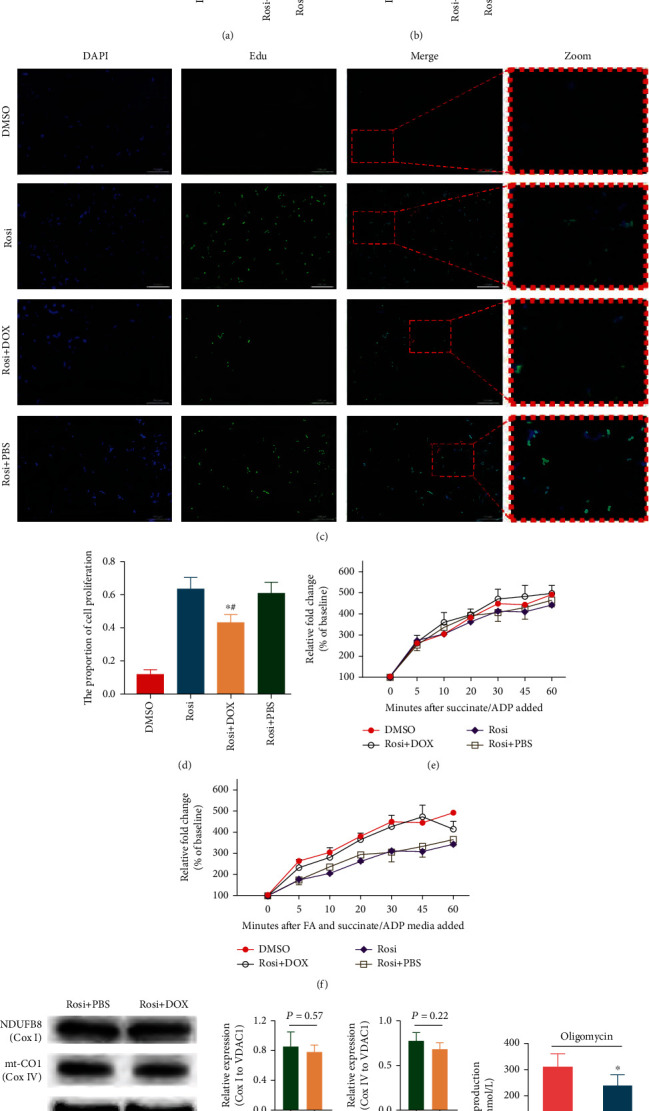
Effects of the forced FOXO1 expression on cell proliferation, mitochondrial OXPHOS, and glycolysis. (a–d) Effects of the forced FOXO1 expression on cell proliferation reflected by CCK-8 measurements (a), ATP production (b), and Edu staining (c, d). The proportions of cell proliferation among four groups were statistically analyzed. DAPI (blue) and Edu (green) represent the nucleus and the newly synthesized DNA, respectively. (e, f) Effects of FOXO1 expression on state 3 respiration in the absence (e) or presence (f) of fatty acid. (g) Ratios of Cox I or IV/VDAC1 were determined by densitometric analysis. Representative immunoblots of Cox I and Cox IV under the forced FOXO1 expression by DOX. VDAC1 served as the loading control. (h) Effect of Rosi on ATP level produced by glycolysis. FOXO1 was induced by DOX to the level similar to that without Rosi. Data represent three independent experiments and are expressed as mean ± SEM (a–c, e–h). ^∗^*P* < 0.05 versus Rosi+PBS; ^#^*P* < 0.05 versus DMSO. FA: fatty acid.

Next, we determined whether the forced FOXO1 expression can counteract the promotion of NSC proliferation by Rosi. The tetracycline-induced expression system was used for the forced expression of FOXO1, and DOX at 1 *μ*L/mL resulted in a similar FOXO1 expression level as that in the DMSO control cells, which was used in the subsequent experiments. The forced FOXO1 expression by DOX was able to counteract the Rosi-induced increase of NSC proliferation, as demonstrated by CCK-8 measurement (Figure 5(a)), ATP production (Figure 5(b)), and Edu incorporation (Figures 5(c) and 5(d)). FOXO1 expression slightly increased state 3 mitochondrial respiration (Figure 5(e)) and greatly increased oxygen consumption in FA media (Figure 5(f)), indicating that FOXO1 increased the mitochondrial respiratory capacity and promoted OXPHOS. However, the main mitochondrial complexes I and IV were not significantly altered by the forced FOXO1 expression (Figure 5(g)). Under oligomycin treatment, there was a decrease in ATP production by glycolysis in FOXO1-expressing NSCs (Figure 5(h)). These results supported the claim that FOXO1 expression was decreased by Rosi and inhibited glycolysis, but it enhanced the mitochondrial oxidation capacity. As expected, the forced FOXO1 expression promoted mitophagy, as indicated by both immunofluorescence colabeled LC3B/Tomm20 and the mitoTimer indicator (Figure S4). This result was concordant with the finding that the respiratory capacity was increased. Still, no change in mitochondrial complex contents was observed since mitophagy is the main mechanism that ensures OXPHOS enzymatic activity.

Taken together, these data demonstrated that the effects of Rosi on the promotion of NSC proliferation and inhibition of PINK1 expression were mediated by the suppression of transcription factor FOXO1 expression rather than other transcription factors involved in TZDs-related pathways.

### 3.4. FOXO1 Directly Binds to the First Intron of PINK1 and Promotes Transcription

To unravel the underlying mechanism by which FOXO1 as a transcription factor regulates PINK1 expression, we studied the role of FOXO1 in the promoter activity of PINK1. The genomic location of the rat PINK1 gene promoter has not yet been identified. The rat PINK1 genomic sequences are located on chromosome 5, spanning 12113 bp (chr5: 156677146 to 156689258). Transcription starts from 156677146 nt, while translation starts from 156677182 nt. We adopted the −2300 bp (chr5: 156675183–156677182) counting from the translation start site, as described in the previous reports [26, 28]. Putative FOXO1 binding sites (FOXO1 consensus sequence (FCS)) inside this sequence were predicted by the GenomatixMatInspector. Unexpectedly, no FCSs were found in the sequences. We continued to explore the potential FCS in the first intron since the first intron is widely believed to regulate gene transcription, and it could be a part of the promoter. It spans a total of 4980 bp (chr5: 156677569–156682548), and three putative FCSs (FCS1, chr5: 156680169–156680185, +3022 bp; FCS2, chr5: 156681222–156681238, +4076 bp; FCS3, chr5: 156681244–156681260, +4098 bp; counting from the translation site) were identified.

**Figure 6:**
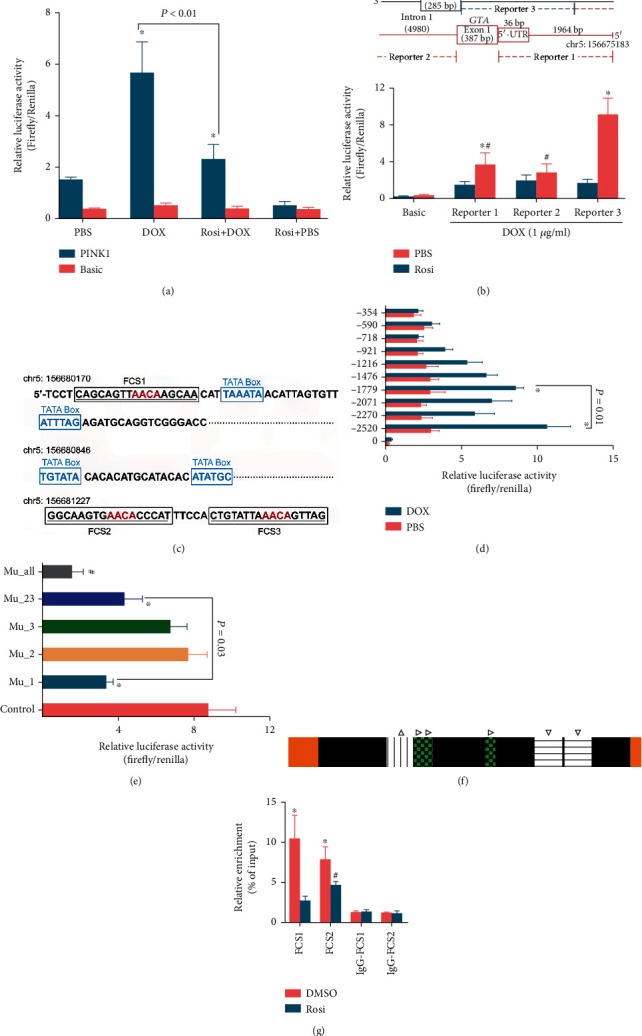
Identification of the core sequences on transcription regulation in the PINK1 gene. (a) Dual-luciferase reporter gene assay results showing region of interest in PINK1 gene increased luciferase activity. The whole region (−2300 bp accounting from the translation site and the first intron) was used. (b) The schematic illustrated three reporters divided from the whole region of interest in PINK1 gene (upper). Dual-luciferase reporter gene assay results showing the effects of the respective reporter in luciferase activity (lower). (c) The schematic illustrating FOXO1 binding sites in the first intron of PINK1 gene predicted by GenomatrixMatInspector. Putative core promoter elements around FOXO1 binding sites were proposed by GPMiner. (d) Dual-luciferase reporter gene assay showing promoter deletion analysis of reporter 3, which contains three FOXO1 binding sites. (e) Effects of FOXO1 binding site mutations on luciferase activity. Control: FOXO1 was induced to expression, and DMSO was added instead of Rosi. (f) The schematic illustrating regulatory elements for transcription in PINK1 gene (upper). Yellow, exons; Black, the first intron. The vertical line, FOXO1 binding site 1 (△); transverse line, FOXO1 binding sites 2 and 3 (▽); green with black blocks inside, TATA box (▷). (g) Results of CHIP analysis using a FOXO1 antibody. Data represent three independent experiments and are expressed as mean ± SEM (a, b, d–f). ^∗^*P* < 0.05 versus basic vector (a, b), the 2371 segment (d), the control (e), or the DMSO treatment (f); ^#^*P* < 0.05 versus basic vector (d) or Mu-1 (e). UTR: untranslated region; FCS: FOXO1 consensus sequences; Mu_1, Mu_2, Mu_3, Mu_23, and Mu_all denote mutations of FCS1, FCS2, FCS3, FCS2/3, and FCS 1/2/3, respectively.

We next validated the effects of these putative regulatory sequences on FOXO1-induced PINK1 expression using the dual-luciferase reporter gene assay. First, both the −2300 bp and the first intron sequences were ligated into the pGL3-Basic vector to evaluate all potential sequences' overall effects. To best mimic the finding that Rosi promotes PINK1 transcription via suppression of FOXO1, we used the inducible expression system and induced FOXO1 expression before Rosi administration to avoid the constitutive expression of FOXO1, which completely abolished the effects of Rosi. The FOXO1 expression was induced for 24 hours in the medium containing DOX (1 *μ*g/mL). NSCs were transfected with luciferase activity reporter vectors and cultured in a tetracycline-free medium containing Rosi (50 *μ*g/mL) for another 24 hours. The results of luciferase activity are summarized in Figure 6(a). The constructed sequences activated transcription, which was augmented by the forced FOXO1 expression. Rosi counteracted this effect in situations with and without the induced FOXO1 expression (Figure 6(a)). This result demonstrated the regulatory function of the whole sequence and directly showed negative regulation of the PINK1 promoter activity by Rosi.

To more precisely identify the sequence response to FOXO1, the whole sequence (−2300 bp counting from the translation start site to the end of the first intron, except exon1) was divided into three reporters (reporter 1: 2300 bp; reporter 2: 2460 bp; reporter 3: 2523 bp; Figure 6(b)), and we examined the effect of each reporter on the PINK1 transcriptional activity. FOXO1 expression was induced before Rosi administration, as stated above. As shown in Figure 6(b), reporter 3 responded to Rosi most obviously, resulting in a 5.39-fold decrease in luciferase activity. Reporters 1 and 2 also responded to Rosi, resulting in a 2.47- and 1.46-fold luciferase activity decrement, respectively (Figure 6(b)). Hence, reporter 3, which contained all three FCSs, was the most important in Rosi-induced repression of PINK1 transcription.

We further divided reporter 3 into nine fragments to analyze the functional sequences in detail, with an approximate 50–100 bp overlap between the fragments to ensure that the core sequences were not destroyed. Primers used were listed in Supplementary Tables. The first FOXO1 consensus sequence (FCS1) was located in the −2523 segment, while the second and third consensus sequences were located in the −1779 segment (Figure 6(c), counting from the end of the first intron). Since there is only a 5 bp distance between the second and the third FOXO1 consensus sequences and that it is not easy to analyze them separately, we analyzed these two sequences (FCS2 and FCS3) as a whole (FCS2). Seven of 10 segments showed increased luciferase activity upon FOXO1 expression (Figure 6(d)). However, unexpectedly, compared with the −1779 segment (FCS2, containing two FOXO1 consensus sequences), the −2523 segment (FCS1, containing one FOXO1 consensus sequence) showed even higher luciferase activity (Figure 6(d)). GPMiner identified distinct distributions of putative core promoter elements around FCS1 and FCS2. Two TATA boxes adjoining the FCS1 were identified, and they were located at 3 bp and 23 bp of the 3′-flank (Figure 6(c), Figure S5). In contrast, the core promoter elements closest to FCS2 were other TATA boxes, located at 381 bp of the 5′-flank (Figure 6(c), Figure S5). Besides the TATA box, no other core promoter element was found within the 500 bp region of both FCS1 and FCS2 (Figure S5).

Site-directed mutagenesis was used to validate the predicted binding sites. All of the predicted binding sites in FCS1 and FCS2 were mutated (FCS1: cagcagtt*AACA*agcaa→cagcagtt*GGTA*agcaa; FCS2: ggcaagtg*AACA*cccat→ggcaagtg-*GGTA*cccat and ctgtatta*AACA*gttag→ctgtatta*GGTA*gttag), and then their transcription activities were compared with those of the wild types. As shown in Figure 6(e), the mutation in FCS1 significantly reduced the luciferase activity, while a mutation in each of the two sites in FCS2 did not significantly affect the transcriptional activity. However, a mutation in both sites in FCS2 resulted in a moderate reduction of the luciferase activity (Figure 6(e)). Notably, the luciferase activity of the mutated FCS1 was lower than that of the mutated FCS2 (Figure 6(e)). Taken together, these data indicated that both FCS1 and FCS2 were involved in the increased PINK1 transcription by FOXO1, but FCS1 was more important.

To confirm that FOXO1 directly binds to the consensus sequences and assess the influence of Rosi, we performed CHIP analysis. FOXO1 expression was induced, and Rosi was administered as previously described. Coimmunoprecipitation with anti-FOXO1 antibody resulted in a positive PCR result using the primer around FOXO1 consensus DNA sequences, while the other primer sets did not yield a positive amplicon. Compared with the DMSO control, Rosi decreased DNA enrichment by anti-FOXO1 protein antibody to approximately 30% (Figure 6(f)). Enrichment of DNA sequences around FCS1 or FCS2 was similar without Rosi, while FCS1 decreased more significantly upon Rosi treatment (Figure 6(f)). The decreased binding capacity may either be because of suppressed FOXO1 expression or due to defects in DNA binding. We used Rosi and DOX (1 *μ*g/mL) together, inducing the constitutive expression of FOXO1 under Rosi treatment to exclude the influence of FOXO1 expression levels on the binding capacity. The CHIP data revealed that Rosi did not significantly affect FCS1 and FCS2 DNA enrichment in the presence of DOX (Figure 6(g)). These results demonstrated that FOXO1 was directly bound to both FCS1 and FCS2 in the first intron of the PINK1 gene. Rosi inhibited this process mainly by decreasing FOXO1 expression rather than by reducing the binding of FOXO1 to the DNA.

## 4. Discussion

Inflammation plays a crucial role in neurodegenerative diseases. For example, chronic flammation is implicated in the pathogenesis of Alzheimer's disease and SCI, and blocking inflammation can effectively alleviate the occurrence of the disease [29]. In the process of spinal cord injury, overactivated inflammatory cascade aggravated neuronal necrosis or apoptosis [30, 31]. So reducing the hyperactive inflammation could be beneficial for neural cell survival and SCI. In this study, we found that Rosi can alleviate the expression of inflammatory cytokines IL-1*β* and IL-6 under SCI, which is consistent with our previous result [32].

NSC proliferation is crucial for the repair of SCI. However, NSCs are the type of cells that proliferate slowly. Therefore, acceleration of the proliferation of NSCs is a matter of considerable importance, and it is also a great challenge in this field. We previously reported the beneficial effects of Rosi [12]. We further explored the underlined physiological mechanism related to mitophagy and NSC proliferation and the detailed molecular mechanism on mitophagy and PINK1 expression regulation in the present study.

In the field of medications for diabetes, metformin, other than TZDs, is the first-line therapy recommended in T2DM guidelines [33]. An *in vitro* study showed the association between metformin usage and neural stem cell proliferation and survival against insults [34–36]. Recent animal studies have also proposed the protective effects of metformin in SCI by inducing nonselective autophagy in neurons [37, 38]. However, there is no direct evidence for metformin *in vivo* proliferation of NSCs is available. In contrast, for TZDs, not only their effects on proliferation in normal rats have been demonstrated in the previous study [39], but they have also been found to be beneficial in recovery from SCI and promotion of proliferation of NSCs in SCI rats in the present study. Thus, our study may indicate the preference for using TZDs in diabetic SCI patients.

To our knowledge, this is the first study related to mitophagy in the biological function of NSCs. Unlike nonselective autophagy, mitophagy is triggered by specific mechanisms, such as PINK1/Parkin, BINP3, NIX, and FUNDC1 pathways. PINK1 expression is crucial for the PINK1/Parkin pathway, which is recognized as the classical trigger and the most important factor in mitophagy, and it plays a role in various disease conditions. Mitophagy serves as a compensatory mechanism upon loss of mitochondrial potential. It is believed to improve mitochondrial function and reduce apoptosis [40]. This seems to be contradictory to the proliferative effects of reduced mitophagy conferred by Rosi observed in our study. However, it should be noted that, in the absence of any other treatment, the membrane potential and ROS production were similar between Rosi- and DMSO-treated NSCs, but enhanced mitophagy was observed in DMSO-treated cells in this situation. This indicates that redundant mitophagy exists in NSCs. Excessive mitophagy leads to neuronal death, and internal environmental disturbance has been observed [41, 42]. Recently, inadequate activation of mitophagy has been reported to cause aberrant homeostasis in some stem cells [43, 44].

Moreover, mitophagy maturation has been recognized as the hallmark of a metabolic substrate switch from fatty acids to glucose [19]. Such a shift may cause a decrease in glucose bioavailability, resulting in a “glucose deprivation-like” effect and impairing proliferation because certain glucose utilization levels have been demonstrated to promote self-renewal of NSCs [45]. Indeed, as found in our work, Rosi resulted in a reduction in oxygen consumption in the presence of fatty acids and an increase in glycolysis, which supported the role of Rosi in maintaining glucose utilization in NSCs. Therefore, although mitophagy is considered a compensatory response, Rosi modulates substrate metabolism and ATP production methods through the regulation of mitophagy, which finally accelerates the proliferation of NSCs.

There has been limited research concerning Rosi and mitophagy. Their associations were presented in two studies, which reported a decrease in mitophagy but the promotion of mitochondrial biogenesis in both adipose and kidney proximal tubular epithelial cells [46, 47], which is in accordance with the findings of the present study. Currently, no research has directly evaluated the association between TZDs and mitophagy in the nervous system except for the present study. However, a previous study showed that Rosi prevented the loss of mitochondrial PINK1 in neuroblastoma [48], which implied decreased mitophagy. Some studies have focused on Rosi and nonselective autophagy. Rosi has been reported to inhibit autophagy in the spinal cord tissue, but no specific cell type was identified [49]. Other studies showed that Rosi induced autophagy in neurons [50]. Therefore, Rosi may contribute to both promotion and inhibition of autophagy depending on the pathological condition [50, 51]. Besides, previous studies on the association of mitochondrial markers supported the effects of Rosi on mitochondrial biogenesis observed in the present study. We observed the increase in red fluorescence and significantly enhanced green fluorescence by mitoTimer, which indicated an increased amount in mitochondria. These results are supported by the previous NSC studies consistently documenting an increased expression of PPAR coactivator-1*α*, a critical transcriptional factor in mitochondrial biogenesis [13–15]. Thus, the present study suggested that mitochondrial clearance and biogenesis could be regulated simultaneously.

In the present study, we characterized the core regulatory sequences involved in the regulation of PINK1 by Rosi. The promoter region of PINK1 in either humans or rodents has not yet been identified. FOXO1 has been reported to bind to the PINK1 promoter and induce transcription. Still, a specific binding site has not been investigated, and the primers used for CHIP resulted in a product exceeding 1600 bp [52]. Hou and colleagues identified the sequence of a 0.4 kbp segment of the DNA immediately 5′-flank to the PINK1 translation start site that bound to FOXO3a in a mouse model [53]. Other studies also identified a segment consisting of 3060 bp (−3061 bp to −1 bp counting from the translation start site) and a segment composed of 1825 bp (−1799 to +26 bp counting from the transcription start site) as the promoter regions of the human PINK1 gene [54, 55]. Currently, to our knowledge, this is the first study to report the sequences involved in the regulation of PINK1 transcription in rats, which could be recognized as a part of the promoter region. We confirmed that the binding site of FOXO1 is located inside the first intron, instead of the regular −2300 bp upstream of the translation start site. This is not surprising since the intron is widely known to participate in the regulation of gene transcription [56–59].

Some limitations of the current study should be acknowledged. First, Rosi has been reported to maintain the undifferentiated phenotypes of NSCs [60], but we did not investigate the differentiation capacity of NSCs, thus leading to a lack of clarity on the potency of NSCs to differentiate into mature neurons. Second, we did not further explore the effect of -directed mutagenesis in PINK1 promoter on NSC proliferation, leading to the unknown of potential NSC proliferation alteration conferred by mutagenesis in PINK1 promoter. Finally, the effects of FOXO1 and PINK1 in Rosi-induced NSCs proliferation were not examined *in vivo*.

**Figure 7:**
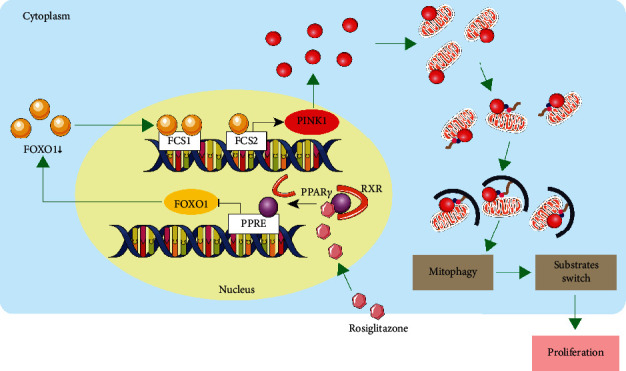
Graphic abstract. The present study showed that the inhibitions of FOXO1 and PINK1 were critically involved in the mechanisms of rosiglitazone-induced mitophagy suppression, which greatly contributed to the enhanced glycolysis and proliferations of NSCs. Besides, we identified that the reduced FOXO1 level, which directly binded to the transcriptional regulation region in PINK1 gene, is the main underlying mechanisms for the decreased PINK1 and mitophagy.

In summary, the present study provided data showing the effects of SCI repairment, inflammation inhibition, NSC proliferation, and mitophagy suppression conferred by Rosi and confirming the causality between mitophagy suppression and NSC proliferation. Moreover, we demonstrated the role of FOXO1 targeting the first intron of PINK1 in Rosi-induced mitophagy inhibition, metabolic substrate shift, and proliferation of NSCs. Furthermore, the regulatory sequences inside the PINK1 promoter region that responded to Rosi and FOXO1 were identified in depth (Figure 7).

## Data Availability

The authors declare that all data supporting the findings of this study are available within the paper and its supplementary information files.

## Supplementary Materials

Supplementary Materials Figure S1: effects of Rosi on oxidative phosphorylation and glycolysis. (A, B) Effects of rosiglitazone on state 2 and state 3 respiration at steady-state. Oxygen consumption was similar during basic state 2 and state 3 respiration between Rosi and DMSO treatments while it was slightly increased by treatments with higher doses of Rosi. (C) Rosi moderately or significantly reduced state 3 respiration under CCCP treatment dependent on the concentration of Rosi. (D) Rosi dramatically reduced state 3 respiration in fatty acid (FA) media, and it reduced approximately half of the oxygen consumption compared with that in the DMSO-treated cells. (E) Results of western blotting for the respiratory complexes showed that Rosi slightly increased the complex I content in the mitochondrion, and it did not affect complex IV expression. VDAC1 was served as the loading control. Data represented three independent experiments and were expressed as mean ± SEM (A–D). ^∗^*P* < 0.05 versus DMSO treatment; ^#^*P* < 0.05 versus 30 *μ*g/mL Rosi (D). ADP: adenosine diphosphate. Figure S2: effects of different rosiglitazone concentrations on supernatant lactate contents. Only a slight increase in lactic acid was observed. Data represented three independent experiments and were expressed as mean ± SEM. Figure S3: effects of Rosi on the mRNA and protein contents of FUNDC1, BNIP3, and NIX in NSCs. The mRNA and protein contents were measured by RT-qPCR (A) and western blotting (B), respectively. *β*-Actin was used as internal reference. Data represented three independent experiments and were expressed as mean ± SEM. Figure S4: effects of the forced FOXO1 expression on Rosi-induced mitophagy suppression. FOXO1 expression was induced by doxycycline (DOX, 1 *μ*g/mL), and the same volume of PBS was used as control. (A) Confocal immunofluorescence images showing LC3B (red) and Tomm20 (green) in NSCs. (B) Confocal images showing the fluorescence of mitoTimer. Rosi was applied in all the groups additionally to the indicated treatments. Scale bar represents 10 *μ*m. Figure S5: results for putative core regulatory element prediction of the first intron in PINK1 gene by the GPMiner method. Table S1: primers for promoter deletion analysis for reporter 3. Table S2: primers for site mutations. Table S3: primers for real-time PCR.

## References

[1] Sas A. R., Carbajal K. S., Jerome A. D. (2020). A new neutrophil subset promotes CNS neuron survival and axon regeneration. *Nature Immunology*.

[2] Paterniti I., Esposito E., Cuzzocrea S. (2014). Phosphodiesterase as a new therapeutic target for the treatment of spinal cord injury and neurodegenerative diseases. *Current Medicinal Chemistry*.

[3] Kuboyama T., Tohda C., Komatsu K. (2014). Effects of Ashwagandha (roots of Withania somnifera) on neurodegenerative diseases. *Biological & Pharmaceutical Bulletin*.

[4] Silva N. A., Sousa N., Reis R. L., Salgado A. J. (2014). From basics to clinical: a comprehensive review on spinal cord injury. *Progress in Neurobiology*.

[5] Karova K., Wainwright J. V., Machova-Urdzikova L. (2019). Transplantation of neural precursors generated from spinal progenitor cells reduces inflammation in spinal cord injury via NF-*κ*B pathway inhibition. *Journal of Neuroinflammation*.

[6] Noori L., Arabzadeh S., Mohamadi Y. (2021). Intrathecal administration of the extracellular vesicles derived from human Wharton's jelly stem cells inhibit inflammation and attenuate the activity of inflammasome complexes after spinal cord injury in rats. *Neuroscience research*.

[7] Younsi A., Zheng G., Scherer M. (2020). Three growth factors induce proliferation and differentiation of neural precursor cells *in vitro* and support cell-transplantation after spinal cord injury *in vivo*. *Stem Cells International*.

[8] Hachem L. D., Mothe A. J., Tator C. H. (2017). Positive modulation of AMPA receptors promotes survival and proliferation of neural stem/progenitor cells from the adult rat spinal cord. *Stem Cells and Development*.

[9] Wang D., Wang K., Liu Z., Wang Z., Wu H. (2021). Valproic acid labeled chitosan nanoparticles promote the proliferation and differentiation of neural stem cells after spinal cord injury. *Neurotoxicity Research*.

[10] Cusimano M., Brambilla E., Capotondo A. (2018). Selective killing of spinal cord neural stem cells impairs locomotor recovery in a mouse model of spinal cord injury. *Journal of Neuroinflammation*.

[11] Shin J. E., Jung K., Kim M. (2018). Brain and spinal cord injury repair by implantation of human neural progenitor cells seeded onto polymer scaffolds. *Experimental & molecular medicine*.

[12] Meng Q.-Q., Lei W., Chen H. (2018). Combined rosiglitazone and Forskolin have neuroprotective effects in SD rats after spinal cord injury. *PPAR Research*.

[13] Chiang M.-C., Nicol C. J., Cheng Y.-C., Lin K. H., Yen C. H., Lin C. H. (2016). Rosiglitazone activation of PPAR*γ*-dependent pathways is neuroprotective in human neural stem cells against amyloid-beta-induced mitochondrial dysfunction and oxidative stress. *Neurobiology of Aging*.

[14] Chiang M. C., Cheng Y. C., Lin K. H., Yen C. H. (2013). PPAR*γ* regulates the mitochondrial dysfunction in human neural stem cells with tumor necrosis factor alpha. *Neuroscience*.

[15] Chiang M.-C., Cheng Y.-C., Nicol C. J., Lin C. H. (2017). The neuroprotective role of rosiglitazone in advanced glycation end product treated human neural stem cells is PPARgamma-dependent. *The International Journal of Biochemistry & Cell Biology*.

[16] Vara-Perez M., Felipe-Abrio B., Agostinis P. (2019). Mitophagy in cancer: a tale of adaptation. *Cells*.

[17] Zhao Y., Huang S., Liu J. (2018). Mitophagy contributes to the pathogenesis of inflammatory diseases. *Inflammation*.

[18] Harris J., Deen N., Zamani S., Hasnat M. A. (2018). Mitophagy and the release of inflammatory cytokines. *Mitochondrion*.

[19] Gong G., Song M., Csordas G., Kelly D. P., Matkovich S. J., Dorn G. W. (2015). Parkin-mediated mitophagy directs perinatal cardiac metabolic maturation in mice. *Science (New York, N.Y.)*.

[20] Zhang H., Menzies K. J., Auwerx J. (2018). The role of mitochondria in stem cell fate and aging. *Development (Cambridge, England)*.

[21] Lange C., Turrero Garcia M., Decimo I. (2016). Relief of hypoxia by angiogenesis promotes neural stem cell differentiation by targeting glycolysis. *The EMBO Journal*.

[22] Zhang J., Liu L., Xue Y. (2018). Endothelial monocyte-activating polypeptide-II induces BNIP3-mediated mitophagy to enhance temozolomide cytotoxicity of glioma stem cells via down-regulating MiR-24-3p. *Frontiers in Molecular Neuroscience*.

[23] Koehler C. L., Perkins G. A., Ellisman M. H., Jones D. L. (2017). Pink1 and Parkin regulate drosophila intestinal stem cell proliferation during stress and aging. *The Journal of Cell Biology*.

[24] Hernandez G., Thornton C., Stotland A. (2013). MitoTimer: a novel tool for monitoring mitochondrial turnover. *Autophagy*.

[25] Mazzone A., Gibbons S. J., Bernard C. E. (2015). Identification and characterization of a novel promoter for the human *ANO1* gene regulated by the transcription factor signal transducer and activator of transcription 6 (STAT6). *FASEB Journal : Official Publication of the Federation of American Societies for Experimental Biology*.

[26] Zhang H.-F., Wu M.-X., Lin Y.-Q. (2017). IL-33 promotes IL-10 production in macrophages: a role for IL-33 in macrophage foam cell formation. *Experimental & Molecular Medicine*.

[27] Silva T. B., Oliveira C. Z., Faria E. F., Mauad E. C., Syrjänen K. J., Carvalho A. L. (2015). Development and validation of a nomogram to estimate the risk of prostate cancer in Brazil. *Anticancer Research*.

[28] Hadji F., Boulanger M.-C., Guay S.-P. (2016). Altered DNA methylation of long noncoding RNA H19 in calcific aortic valve disease promotes mineralization by silencing NOTCH1. *Circulation*.

[29] Holmes C. (2013). Review: systemic inflammation and Alzheimer’s disease. *Neuropathology and Applied Neurobiology*.

[30] Ahuja C. S., Fehlings M. (2016). Concise review: bridging the gap: novel neuroregenerative and neuroprotective strategies in spinal cord injury. *Stem Cells Translational Medicine*.

[31] Zhang B., Gensel J. C. (2014). Is neuroinflammation in the injured spinal cord different than in the brain? Examining intrinsic differences between the brain and spinal cord. *Experimental Neurology*.

[32] Meng Q.-Q., Feng Z.-C., Zhang X.-L. (2019). PPAR-*γ* Activation Exerts an Anti-inflammatory Effect by Suppressing the NLRP3 Inflammasome in Spinal Cord-Derived Neurons. *Mediators of Inflammation*.

[33] Kalyani R. R., Cannon C. P., Cherrington A. L. (2018). Professional Practice Committee: *Standards of Medical Care in Diabetes-2018*. *Diabetes Care*.

[34] Fatt M., Hsu K., He L. (2015). Metformin acts on two different molecular pathways to enhance adult neural precursor proliferation/self-renewal and differentiation. *Stem Cell Reports*.

[35] Chung M.-M., Nicol C. J., Cheng Y.-C. (2017). Metformin activation of AMPK suppresses AGE-induced inflammatory response in hNSCs. *Experimental Cell Research*.

[36] Lin C.-H., Cheng Y.-C., Nicol C. J., Lin K. H., Yen C. H., Chiang M. C. (2017). Activation of AMPK is neuroprotective in the oxidative stress by advanced glycosylation end products in human neural stem cells. *Experimental Cell Research*.

[37] Guo Y., Wang F., Li H. (2018). Metformin protects against spinal cord injury by regulating autophagy via the mTOR signaling pathway. *Neurochemical Research*.

[38] Wang P., Xie Z.-D., Xie C.-N. (2018). AMP-activated protein kinase-dependent induction of autophagy by erythropoietin protects against spinal cord injury in rats. *CNS Neuroscience & Therapeutics*.

[39] Morales-Garcia J. A., Luna-Medina R., Alfaro-Cervello C. (2011). Peroxisome proliferator-activated receptor *γ* ligands regulate neural stem cell proliferation and differentiation in vitro and in vivo. *Glia*.

[40] Pickles S., Vigié P., Youle R. J. (2018). Mitophagy and quality control mechanisms in mitochondrial maintenance. *Current biology : CB*.

[41] Shi R., Weng J., Zhao L., Li X. M., Gao T. M., Kong J. (2012). Excessive autophagy contributes to neuron death in cerebral ischemia. *CNS Neuroscience & Therapeutics*.

[42] Yu E. P. K., Reinhold J., Yu H. (2017). Mitochondrial respiration is reduced in atherosclerosis, promoting necrotic core formation and reducing relative fibrous cap thickness. *Arteriosclerosis, Thrombosis, and Vascular Biology*.

[43] Jin G., Xu C., Zhang X. (2018). Atad3a suppresses Pink1-dependent mitophagy to maintain homeostasis of hematopoietic progenitor cells. *Nature Immunology*.

[44] Yang C., Suda T. (2018). Hyperactivated mitophagy in hematopoietic stem cells. *Nature Immunology*.

[45] Fusco S., Leone L., Barbati S. A. (2016). A CREB-Sirt1-Hes1 circuitry mediates neural stem cell response to glucose availability. *Cell Reports*.

[46] Small D. M., Morais C., Coombes J. S., Bennett N. C., Johnson D. W., Gobe G. C. (2014). Oxidative stress-induced alterations in PPAR-*γ* and associated mitochondrial destabilization contribute to kidney cell apoptosis. *American journal of physiology Renal physiology*.

[47] Taylor D., Gottlieb R. A. (2017). Parkin-mediated mitophagy is downregulated in browning of white adipose tissue. *Obesity (Silver Spring, Md)*.

[48] Corona J. C., de Souza S. C., Duchen M. R. (2014). PPAR*γ* activation rescues mitochondrial function from inhibition of complex I and loss of PINK1. *Experimental Neurology*.

[49] Li H., Zhang Q., Yang X., Wang L. (2017). PPAR-*γ* agonist rosiglitazone reduces autophagy and promotes functional recovery in experimental traumaticspinal cord injury. *Neuroscience Letters*.

[50] Zhao Z., Zhang L., Guo X.-D. (2017). Rosiglitazone exerts an anti-depressive effect in unpredictable chronic mild-stress-induced depressive mice by maintaining essential neuron autophagy and inhibiting excessive astrocytic apoptosis. *Frontiers in Molecular Neuroscience*.

[51] Shao Z.-Q., Liu Z.-J. (2015). Neuroinflammation and neuronal autophagic death were suppressed via rosiglitazone treatment: new evidence on neuroprotection in a rat model of global cerebral ischemia. *Journal of the Neurological Sciences*.

[52] Li W., du M., Wang Q. (2017). FoxO1 promotes mitophagy in the podocytes of diabetic male mice via the PINK1/Parkin pathway. *Endocrinology*.

[53] Mei Y., Zhang Y., Yamamoto K., Xie W., Mak T. W., You H. (2009). FOXO3a-dependent regulation of Pink1 (Park6) mediates survival signaling in response to cytokine deprivation. *Proceedings of the National Academy of Sciences of the United States of America*.

[54] Duan X., Tong J., Xu Q. (2014). Upregulation of human PINK1 gene expression by NF*κ*B signalling. *Molecular Brain*.

[55] Murata H., Takamatsu H., Liu S., Kataoka K., Huh N. H., Sakaguchi M. (2015). NRF2 regulates *PINK1* expression under oxidative stress conditions. *PLoS One*.

[56] Tomaras G. D., Foster D. A., Burrer C. M., Taffet S. M. (1999). ETS transcription factors regulate an enhancer activity in the third intron of TNF-alpha. *Journal of Leukocyte Biology*.

[57] Jin H., van't Hof R. J., Albagha O. M. E., Ralston S. H. (2009). Promoter and intron 1 polymorphisms of COL1A1 interact to regulate transcription and susceptibility to osteoporosis. *Human Molecular Genetics*.

[58] Heyn P., Kalinka A. T., Tomancak P., Neugebauer K. M. (2015). Introns and gene expression: cellular constraints, transcriptional regulation, and evolutionary consequences. *BioEssays : news and reviews in molecular, cellular and developmental biology*.

[59] Laxa M., Müller K., Lange N., Doering L., Pruscha J. T., Peterhänsel C. (2016). The 5'UTR intron of Arabidopsis GGT1 aminotransferase enhances promoter activity by recruiting RNA polymerase II. *Plant Physiology*.

[60] Bernal C., Araya C., Palma V., Bronfman M. (2015). PPARÎ²/Î´ and PPARÎ³ maintain undifferentiated phenotypes of mouse adult neural precursor cells from the subventricular zone. *Frontiers in Cellular Neuroscience*.

