# Supplementary material for: Rosiglitazone Ameliorates Spinal Cord Injury via Inhibiting Mitophagy and Inflammation of Neural Stem Cells
Source: Oxid Med Cell Longev. 2022 Jan 4;2022:5583512. doi: 10.1155/2022/5583512 (PMC8752267; doi:10.1155/2022/5583512)
Supplement: Supplementary Materials — Figure S1: effects of Rosi on oxidative phosphorylation and glycolysis. (A, B) Effects of rosiglitazone on state 2 and state 3 respiration at steady-state. Oxygen consumption was similar during basic state 2 and state 3 respiration between Rosi and DMSO treatments while it was slightly increased by treatments with higher doses of Rosi. (C) Rosi moderately or significantly reduced state 3 respiration under CCCP treatment dependent on the concentration of Rosi. (D) Rosi dramatically reduced state 3 respiration in fatty acid (FA) media, and it reduced approximately half of the oxygen consumption compared with that in the DMSO-treated cells. (E) Results of western blotting for the respiratory complexes showed that Rosi slightly increased the complex I content in the mitochondrion, and it did not affect complex IV expression. VDAC1 was served as the loading control. Data represented three independent experiments and were expressed as mean ± SEM (A–D). ∗P < 0.05 versus DMSO treatment; #P < 0.05 versus 30 μg/mL Rosi (D). ADP: adenosine diphosphate. Figure S2: effects of different rosiglitazone concentrations on supernatant lactate contents. Only a slight increase in lactic acid was observed. Data represented three independent experiments and were expressed as mean ± SEM. Figure S3: effects of Rosi on the mRNA and protein contents of FUNDC1, BNIP3, and NIX in NSCs. The mRNA and protein contents were measured by RT-qPCR (A) and western blotting (B), respectively. β-Actin was used as internal reference. Data represented three independent experiments and were expressed as mean ± SEM. Figure S4: effects of the forced FOXO1 expression on Rosi-induced mitophagy suppression. FOXO1 expression was induced by doxycycline (DOX, 1 μg/mL), and the same volume of PBS was used as control. (A) Confocal immunofluorescence images showing LC3B (red) and Tomm20 (green) in NSCs. (B) Confocal images showing the fluorescence of mitoTimer. Rosi was applied in all the groups additionally to the ind [file 5583512.f1.pdf]

Supplementary materials

Figure S1

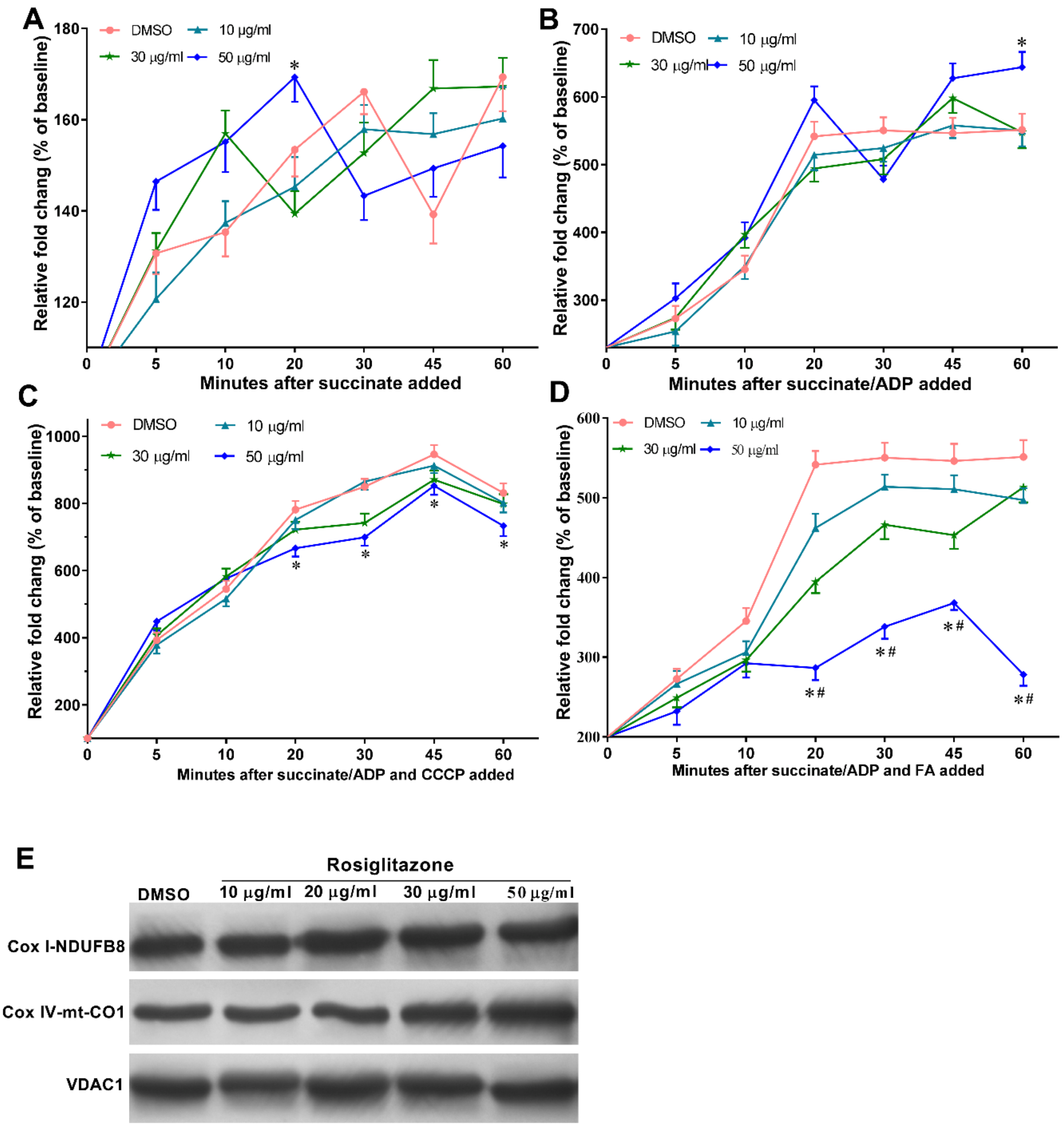

Figure S1. Effects of Rosi on oxidative phosphorylation and glycolysis. A-B, Effects of rosiglitazone on state 2 and state 3 respiration at steady-state. Oxygen consumption was similar during basic state 2 and state 3 respiration between Rosi and DMSO treatments while it was slightly increased by treatments with higher doses of Rosi. **C**, Rosi moderately or significantly reduced state 3 respiration under CCCP treatment dependent on the concentration of Rosi. **D**, Rosi dramatically reduced state 3 respiration in fatty acid (FA) media, and it reduced

approximately half of the oxygen consumption compared with that in the DMSO-treated cells. **E**, Results of western bloting for the respiratory complexes showed that Rosi slightly increased the complex I content in the mitochondrion, and it did not affect complex IV expression. VDAC1 was served as the loading control. Data represented three independent experiments and were expressed as mean±SEM (A-D). **P*<0.05 versus DMSO treatment; #*P*<0.05 versus 30 μg/ml Rosi (D). ADP, adenosine diphosphate.

Figure S2

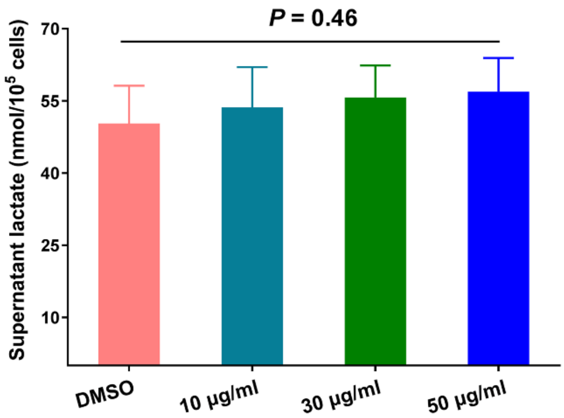

Figure S2. Effects of different rosiglitazone concentrations on supernatant lactate contents. Only a slight increase in lactic acid was observed. Data represented three independent experiments and were expressed as mean±SEM.

Figure S3

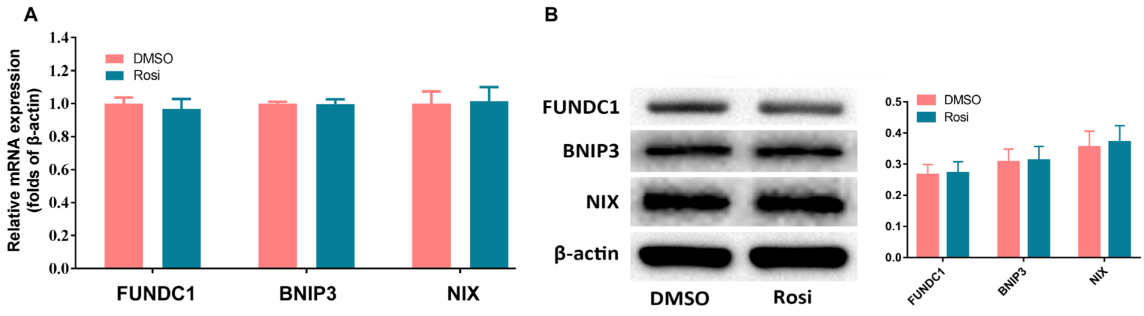

Figure S3. Effects of Rosi on the mRNA and protein contents of FUNDC1, BNIP3, and

NIX in NSCs. The mRNA and protein content were measured by RT-qPCR (**A**) and western blotting (**B**), respectively. β-actin was used as internal reference. Data represented three independent experiments and were expressed as mean±SEM.

Figure S4

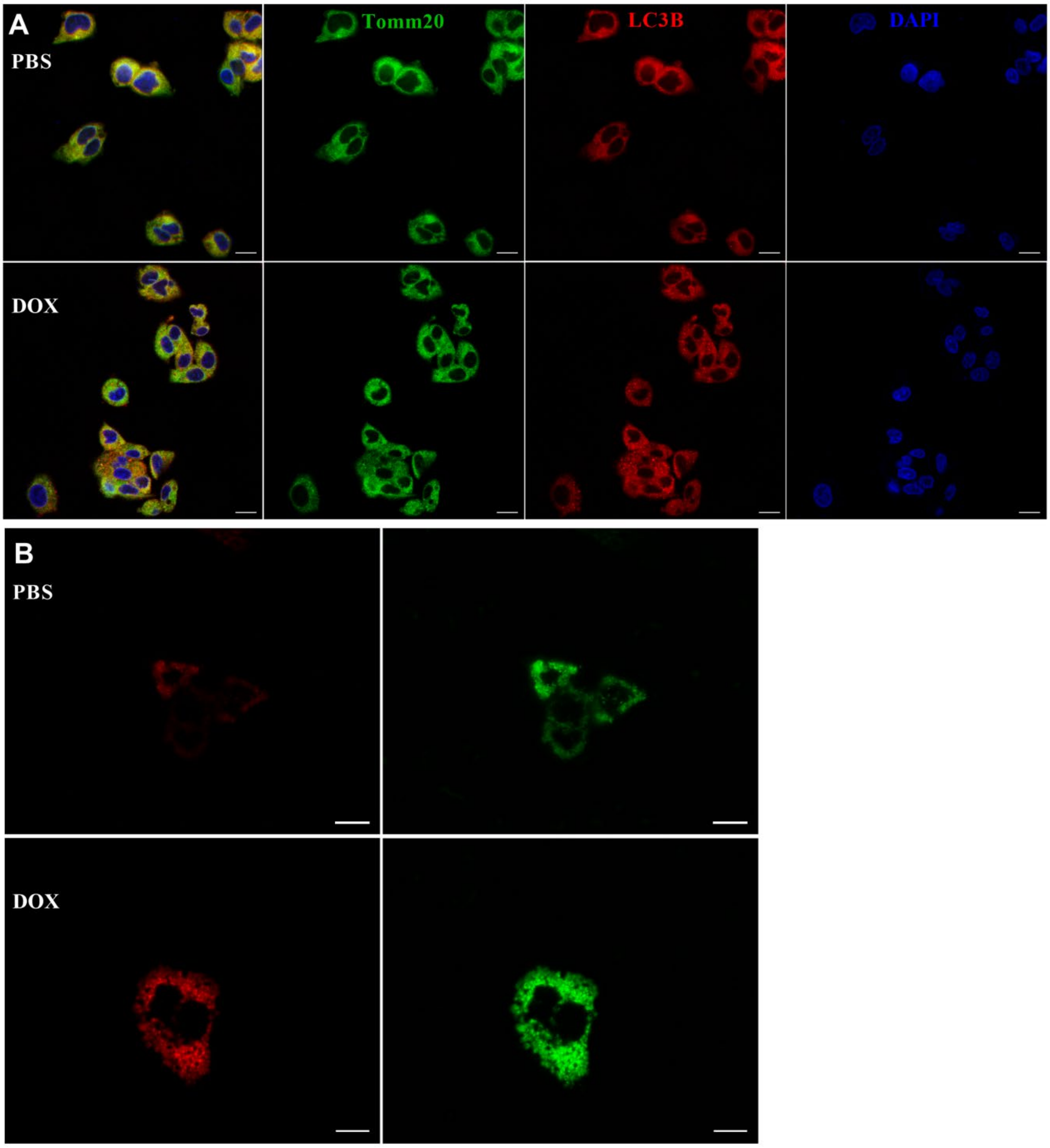

Figure S4. Effects of the forced FOXO1 expression on Rosi-induced mitophagy suppression. FOXO1 expression was induced by doxycycline (DOX, 1 μg/ml) and the same volume of PBS was used as control. **A**, Confocal immunofluorescence images showing LC3B

(red) and Tomm20 (green) in NSCs. **B**, Confocal images showing the fluorescence of mitoTimer. Rosi was applied in all the groups additionally to the indicated treatments. Scale bar represent 10 μ m.

Figure S5

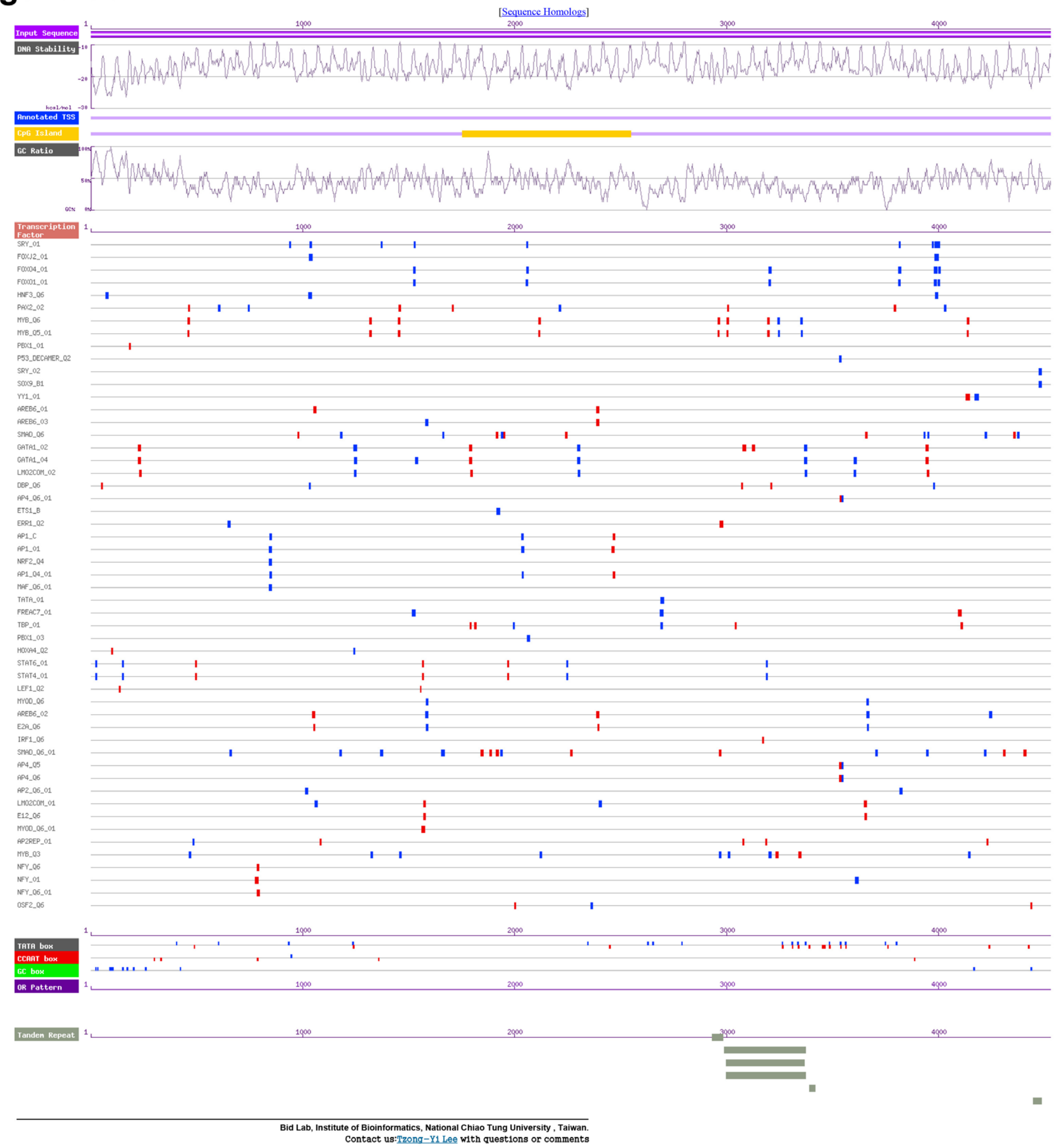

Figure S5. Results for putative core regulatory elements prediction of the first intron in PINK1 gene by the GPMiner method.

Table S1. Primers for promoter deletion analysis for Reporter 3

Annotations	Primers Sequences	Product Length (bp)
-2520	5'-GGCCTCGAGTTACAATTTAAATATAAATCTA-3'	2520
-2270	5'-GGCCTCGAGCTACAAGCTGCACGGATT-3'	2270
-2071	5'-GGCCTCGAGACAAACGCACTCACACAT-3'	2071
-1779	5'-GGCCTCGAGCATACACACATGCACACATA-3'	1779
-1476	5'-GGCCTCGAGGGGTCGTGTACTATGCTAAA-3'	1476
-1216	5'-GGCCTCGAGGGTTATGGAGGAAGGATGAC-3'	1216
-921	5'-GGCCTCGAGTGGTGGACCATACCTCTC-3'	921
-718	5'-GGCCTCGAGGAGTCACTTGTCTTGCTTAG-3'	718
-590	5'-GGCCTCGAGGGTCCACAGAGCAAGTTC-3'	590
-354	5'-GGCCTCGAGAGTTATAGATGGTCGTGATGG-3'	354
Anti-sense	5'-GGCAAGCTTTGAGGGAGAAGAATGCATTG-3'	-

Anti-sense was the same in all clones. Incision enzymes *Xho* I and *Hin*d III were used.

Table S2. Primers for site mutations

Annotations	Primers Sequence
FCS1	sense: 5'-ATAACACTAATGTTATTTAATGTTGCTTACCAACTGCTGAGGAAGACTTCATATGGATACAG-3'
	anti-sense: 5'-CTGTATCCATATGAAGTCTTCCTCAGCAGTTGGTAAGCAACATTAAATAACATTAGTGTTAT-3'
FCS2	sense: 5'-ACTGTTTAATACAGTGGAAATGGGTACCCACTTGCCAATAGCTTCACAAACC-3'
	anti-sense: 5'-GGTTTGTGAAGCTATTGGCAAGTGGGTACCCATTTCCACTGTATTAAACAGT-3'
FCS3	sense: 5'-CCCTAGAAGCAAAGCTCCTAACTACCTAATACAGTGGAAATGGGTGTTCA-3'
	anti-sense: 5'-TGAACACCCATTTCCACTGTATTAGGTAGTTAGGAGCTTTGCTTCTAGGG-3'

Table S3. Primers for real-time PCR

Gene Name	Primers Sequence
STAT3	sense: 5'-GCCTGGTGTGAACTACTCAG-3'<br>anti-sense: 5'-CTGCTGCTTGGTATATGGTCT-3'
NF-κB	sense: 5'-GCCTGGTGTGAACTACTCAG-3'<br>anti-sense: 5'-CTGCTGCTTGGTATATGGTTC-3'
PPAR-α	sense: 5'-GAAGCCTACCTGAAGAACTT-3'<br>anti-sense: 5'-GAGGACAGCATCGTGAAG-3'
PGC-1α	sense: 5'-AAGATGCCTCCTGTGACTGG-3'<br>anti-sense: 5'-GATGACCGAAGTGCTTGTT-3'
LXR	sense: 5'-CTCATAGCCATCAGCATCTTT-3'<br>anti-sense: 5'-TAGGAAGCAGTCAGTAAGCT-3'
CREB-1	sense: 5'-AGGAGTCTGTGGATAGTGTA-3'<br>anti-sense: 5'-TGTGCGAATCTGGTATGTT-3'
CRTC2	sense: 5'-GATGGCGAGATGGATGCTAA-3'<br>anti-sense: 5'-GGAAGTGTAGGTTGGTGAGGT-3'
FOXO1	sense: 5'-GGACGGAGATACCTTGGATT-3'<br>anti-sense: 5'-AATGTTGCCTGCTCACTAACT-3'
β-Actin	sense: 5'-CCATTGAACACGGCATTGTC-3'<br>anti-sense: 5'-TACGACCAGAGGCATACAG-3'
FCS1*	sense: 5'-AAGTCTTCCTCAGCAGTTAA-3'<br>anti-sense: 5'-CCCGAGTTTCTACATTTAGC-3'
FCS2*	sense: 5'-CCTGGTCGATGGTGATTAC-3'<br>anti-sense: 5'-CGTCCCTAGAAGCAAAGC-3'
FUNDC1	sense: 5'-CTTCTACAGGTTGCCAGTCAC-3'<br>anti-sense: 5'-GCCTTATTTGCTCGCTTCTTA-3'
BNIP3	sense: 5'-TCAGCAATGGGAATGGGAGCAG-3'<br>anti-sense: 5'-TGTGGTGTCTGGGAGCGAGGTG -3'
NIX	sense: 5'-CACAAGAAGACGGGCAAATAA-3'<br>anti-sense: 5'-CACGCTTAGGGTGTCTGAAAA-3'

*, These primers were used for CHIP. Annotations of the gene names are listed in Figure 6.
